# Identifying segment-specific barriers to ordering environmentally sustainable plant-based meat dishes in restaurants

**DOI:** 10.1080/09669582.2024.2342982

**Published:** 2024-04-22

**Authors:** David Fechner, Bettina Grün, Sara Dolnicar

**Affiliations:** a Social Marketing Griffith, Business School, Griffith University, Brisbane, Australia; b Institute for Statistics and Mathematics, WU Vienna University of Economics and Business, Vienna, Austria; c Faculty of Business, Economics and Law, The University of Queensland, Business School, St Lucia, Queensland, Australia

**Keywords:** Plant-based meat, capability-opportunity-motivation-behaviour model, restaurant, sustainability, segmentation

## Abstract

Eating less meat when dining out can help mitigate climate change. Plant-based meats can facilitate the transition to a more environmentally sustainable tourism sector. However, uptake of these products remains low. Building on the capability-opportunity-motivation behaviour model, this study identifies the main reasons for the general population of restaurant patrons to reject plant-based meats: they prefer meat and traditional vegetable dishes; they are concerned about not enjoying plant-based meat dishes; they perceive plant-based meat dishes as too expensive. Accounting for heterogeneity among diners leads to the identification of six distinct consumer segments, which differ in their reasons for not ordering plant-based meat dishes in restaurants. From these empirical insights, we derive recommendations for tourism professionals on how to entice specific consumer segments to order plant-based meat dishes and identify future avenues for research.

## Introduction

The “*world is losing the fight against climate change*” (United Nations Environment Programme, 2021). A major contributor to climate change is the global food system, with the expansion of animal agriculture alone threatening the achievement of the Paris Agreement (Clark et al., 2020). Producing meat and dairy is responsible for 56% of food-related greenhouse gases, but provides only 37% and 18% of the total protein and calories (Poore & Nemecek, 2018). The Intergovernmental Panel on Climate Change recommends, therefore, a systematic shift from diets high in meat to plant-dominant diets (Nabuurs et al., 2022).

Given the trend of dining out more (Saksena et al., 2018) and the fact that people eat more meat when dining out than they do at home (Biermann & Rau, 2020), plant-dominant restaurant menus can help create a more environmentally sustainable food system (Fechner et al., 2023). One approach to reducing meat consumption, while still offering consumers a similar experience, is to introduce plant-based meats. Plant-based meats are made from ingredients such as vegetables, mushrooms, or wheat and mimic meat in taste, texture, and smell (Kumar et al., 2017). An emergent body of literature suggests that, on average, plant-based meat has a lower environmental footprint compared to animal meat because it requires less land, water, nitrogen, and emits fewer greenhouse gas emissions (Bryant, 2022; Eshel et al., 2019; Kozicka et al., 2023; Mason-D’Croz et al., 2022; Mazac et al., 2022; Saget et al., 2021; Santo et al., 2020).

Simply replacing meat with plant-based meat may, however, not lead to a just transition (Mouat et al., 2019). A just transition towards more plant-dominant restaurant menus is complex and requires the consideration of environmental, economic, and social ramifications. Substituting a proportion of meat with plant-based meat could, for example, threaten jobs in the animal agriculture (Mason-D’Croz et al., 2022) and regional tourism industry, lead to a sense of identity loss among farmers (Murphy et al., 2022), and threaten the preservation of the cultural heritage of food.

Creating more plant-dominant dishes requires, therefore, the consideration and support of different stakeholder groups, including consumers who are willing to order plant-based meat dishes. To date, uptake of plant-based meats by guests dining out remains, however, low (Clark & Bogdan, 2019; Joseph et al., 2020). Some of the barriers—factors that obstruct or prevent the consumption of plant-based meat dishes (McDonagh et al., 2018)—identified in the home context include taste, healthiness, price, lack of familiarity, and social acceptance of plant-based meats, and preference for traditional vegetable dishes (Jahn et al., 2021; Onwezen et al., 2021). In the context of plant-based meat consumption barriers at home, heterogeneity exists between consumer groups. For example, while meat lovers do not eat plant-based meat because they do not like its taste, consumers who occasionally eat plant-based meat do not cook with these products more frequently because of their unfamiliarity and nutritional profile (Knaapila et al., 2022). This body of literature provides plant-based meat manufacturers with insights on how to promote their products in supermarkets. Strategies include highlighting the health benefits of meat alternatives and showcasing images of the prepared product on the packaging to increase familiarity (Brooker et al., 2022).

We extend the investigation to the restaurant context because initial evidence suggests that restaurants can play an important role in negotiating plant-based meat consumption barriers, by, for example, serving plant-based meat dishes as the default option (Taufik et al., 2022) where reducing the price may not be sufficient to change ordering behaviour (Lemken & Langen, 2023). Research to date has not, however, generated the level of understanding of reasons for consumer resistance required for practitioners to put in place targeted measures to increase uptake of plant-based meats; nor has it investigated heterogeneity in uptake barriers among diners.

The present study fills this gap; specifically, we ask two research questions (What are the dominant barriers preventing restaurant guests from ordering plant-based meat dishes? What restaurant guest segments exhibit distinct barrier patterns?) and provide theory-based recommendations for practitioners on how to overcome the key barriers and entice specific consumer groups to order plant-based meat dishes. In so doing, the present study makes several contributions to knowledge by extending prior work, which was either set in the home context or investigated the effectiveness of individual initiatives in dining contexts, to identifying a comprehensive set of barriers preventing consumers to order plant based meats in restaurants at the aggregate population level and by extracting and profiling consumer segments based on their complete barrier pattern (as opposed to single barriers). We propose future research questions that need to be investigated to effectively leverage consumer heterogeneity to entice specific segments of consumers to order plant-based meats. Importantly, we conduct this study for the general population, rather than for those consumers who have already embraced plant-based meats, because the aim is to entice the general population to convert, at least partially, to ordering plant-based meats in restaurants.

## Literature review

### A just transition towards plant-dominant restaurant menus

A just transition towards plant-dominant restaurant menus is complex and requires the consideration of environmental, economic, and societal ramifications (Kaljonen et al., 2021). We critically assess potential consequences which are specifically relevant to the tourism sector: food-related greenhouse gas emissions of tourism businesses, the livelihoods of people working in the meat production and regional tourism sector, preservation of cultural heritage of food, and nutritional value of restaurant dishes.

A variety of plant-based meat products have entered the market since the early 2000s (Vallikkadan et al., 2023). The potential environmental benefits of replacing animal with plant-based meat depend largely on two factors: what type of meat is being replaced and the ingredients of plant-based meat (e.g., Rubio et al., 2020; Santo et al., 2020). The median greenhouse gas emissions of plant-based alternatives to chicken, pork, and beef are 43%, 63%, and 93% lower than animal meat (Santo et al., 2020). Replacing meat with plant-based alternatives, predominantly made from tofu, soybeans, peanuts, and lentils, could reduce land and nitrogen use, and greenhouse gas emissions of the food system in the United States by 35%–50% (Eshel et al., 2019). Even replacing only 60% of beef with plant-based alternatives made from grains, legumes, oilseeds, and starches could reduce greenhouse gas emissions of the agriculture sector in the United States by 13.5% (Mason-D’Croz et al., 2022). Globally, substituting 50% of pork, chicken, beef and milk with plant-based alternatives, primarily made from soy, rapeseed, potatoes, and beans, could reduce food-related greenhouse gas emissions by 31%, with replacing beef accounting for half of the reduction (Kozicka et al., 2023). However, using mycoprotein, a protein derived from fungi, to replace animal products has shown mixed results: substituting mycoprotein for ruminant meat could significantly reduce the environmental footprint of the agricultural industry (Humpenöder et al., 2022) but not as a replacement for pork or chicken (Souza Filho et al., 2019). Overall, the existing body of literature suggests that replacing a proportion of meat, particularly ruminant meat, with certain plant-based alternatives can help create a more environmentally sustainable food system (Bunge et al., 2022).

To achieve a just transition to plant-dominant restaurant menus, securing people’s livelihoods who are affected by the changes is important (Kaljonen et al., 2021). A significant reduction in demand for meat would threaten jobs in the livestock (Kuhmonen & Siltaoja, 2022; Lehtonen et al., 2022) and regional tourism industry. Substituting a proportion of beef with plant-based alternatives in the United States would threaten up to 45% of jobs in the livestock industry (Mason-D’Croz et al., 2022). Especially, in areas where livestock farmers rely on tourism businesses to purchase their products, a transition to plant-dominant restaurant menus may create economic injustice. Lower demand for animal products could also disrupt agritourism businesses which often rely on livestock to attract tourists to rural areas (Jęczmyk et al., 2021). Plant-based meat products are also often more expensive compared to animal meat which increases cost for tourism businesses and tourists (Lemken & Langen, 2023). Identifying ways to compensate stakeholder groups impacted by the transition to plant-based meat is particularly important because private food companies, the primary driving force behind the shift, prioritise short term return on investments (Guthman et al., 2022) rather than equitable compensations.

A just transition to plant-dominant restaurant menus also needs to consider the meaning of meat in different cultures (Kaljonen et al., 2021) because food is recognised as an intangible cultural heritage (Dembedza et al., 2022). The process of producing, preparing, and consuming food can express cultural practices and values of communities and form people’s individual and social identity (Dembedza et al., 2022). Meat dishes play an important role for many communities to express their culture and attract tourists (Sexton et al., 2019). Replacing traditional meat dishes with plant-based meat may, therefore, threaten the preservation of the intangible cultural heritage of eating meat (Sexton et al., 2019). Furthermore, people who have worked with meat, such as livestock farmers, may experience a loss of identity and mental health issues if they are unable to produce meat (Murphy et al., 2022).

Providing nutritious food is also an important part of a just transition (Kaljonen et al., 2021). Due to the continued growth of the tourism sector, restaurants have been encouraged to offer healthy dishes to help mitigate the global obesity crisis (Seo & Lee, 2017). While initial evidence suggests that plant-based meat can provide the same nutritional value as meat (Eshel et al., 2019), the long-term health effects are uncertain (Toh et al., 2022). The healthiness of plant-based meat depends on the ingredients and certain plant-based meat products can have higher sodium levels and lower protein amounts compared to meat (Tay et al., 2023). Overall, plant-based meat can play a role in creating a more environmentally sustainable food system that produces culturally appropriate and healthy foods at an affordable price if the right measures are in place. The potential benefits of plant-based meat can only be realised if consumers are willing to order these dishes but uptake remains low to date (Clark & Bogdan, 2019; Joseph et al., 2020).

### Capability-opportunity-motivation behaviour model and plant-based meat consumption barriers for restaurant guests

We use the capability-opportunity-motivation behaviour (COM-B) model as the theoretical foundation to summarise the existing body of literature on why consumers are not willing to reduce their meat consumption to propose potential barriers that may prevent consumers from ordering plant-based meat dishes in restaurants. The COM-B model proposes that consumers experience barriers related to their capability, motivation, and opportunity to perform certain behaviours (Michie et al., 2011). Capability describes consumers’ physical and psychological ability to engage in the target behaviour and includes their knowledge, attention, and decision-making ability. Opportunity refers to the social and physical context in which the behaviour occurs. Important factors that reduce the opportunity of consumers to engage in the behaviour include the financial cost and social acceptance of the behaviour. Motivation refers to conscious and unconscious processes that drive the behaviour and include consumers’ beliefs and attitudes, identity, and habits (Michie et al., 2011). We build on different disciplines to provide insights into the biological, psychological, and social factors contributing to the hesitancy of ordering plant-based meat dishes, including evolutionary biology, gender studies, hedonic psychology, identity theory, and sociology.

**Figure 1.**
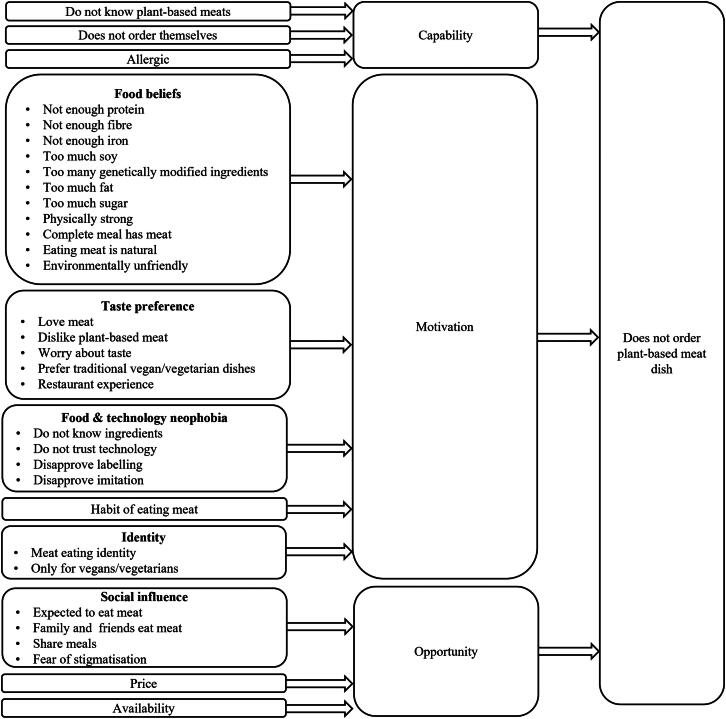
Potential barriers preventing restaurant guests from ordering plant-based meat dishes based on the COM-B model.

Figure 1 summarises the proposed barriers to plant-based meat consumption in restaurants based on the existing literature on why consumers are not willing to adopt a vegetarian or vegan diet or eat plant-based meats at home. Factors reducing the capability to order plant-based meat dishes may include not knowing that plant-based meat dishes are available, not being responsible for deciding what dish is being ordered or being allergic to certain ingredients.

Factors reducing motivation to order plant-based meat dishes may include taste preferences, food beliefs, food neophobia, and identity. Dining out is a special occasion for many restaurant guests and eating a delicious meal is a crucial component to the overall experience (Liu & Tse, 2018). The desire for tasty dishes is grounded in hedonic psychology (Kahneman et al., 1999) which states that consumers seek dishes that provide immediate enjoyment rather than long-term benefits. Many consumers believe meat dishes taste better than plant-based options (Bryant, 2019; Corrin & Papadopoulos, 2017; Elzerman et al., 2013; Hoek et al., 2011b; Kerslake et al., 2022; Michel et al., 2021; Pohjolainen et al., 2015). A taste preference for meat serves a biological function because meat is an easily digestible, calorie-dense source of protein, which was critical for the survival of human ancestors (Breslin, 2013). Standard restaurant practices, such as describing meat dishes with more indulgent language (Turnwald et al., 2020) and labelling meat as pork and beef to dissociate it from its animal origin to prevent feelings of disgust (Benningstad & Kunst, 2020), reinforce the belief that meat is tastier than plant-based options.

Consumers may also lack the motivation to order plant-based meat dishes because they think these products lack important nutrients, such as protein and iron (Collier et al., 2021; Corrin & Papadopoulos, 2017; Kemper & White, 2021; Michel et al., 2021), and contain too much sugar, sodium, saturated fat, soy, and genetically modified ingredients (Collier et al., 2021; Jahn et al., 2021; Kerslake et al., 2022). Although plant-based meat manufacturers promote their products as healthy, the livestock industry created a counternarrative in which plant-based meats are displayed as highly processed, unhealthy foods (Sexton et al., 2019), and in so doing, reinforced the existing belief that meat is necessary for optimal health. When presented with plant-based meat options, consumers may experience food neophobia, a reluctance towards eating unfamiliar foods (Hoek et al., 2011b). Food neophobia was an important survival mechanism for human ancestors because it prevented the consumption of potentially harmful foods (Martins & Pliner, 2005). In a restaurant environment, consumers may be hesitant to ordering unfamiliar plant-based meat dishes because they do not want to risk ordering a dish they will not enjoy (Knaapila et al., 2022).

Not all food choices are conscious decisions, instead, restaurant guests order meat dishes out of habit (de Boer et al., 2017). Habitual behaviours require a cue to be activated (Wood & Rünger, 2016). Common menu design approaches which draw attention to meat dishes may function as trigger to activate the habitual ordering process. Examples include promoting meat dishes as ‘Chef’s Special’, listing them at the top or the bottom of the menu, or serving meat dishes as the default option.

Food-related identities may also reduce consumers’ motivation to order plant-based meat dishes. Many people think of themselves as a meat eater and believe plant-based foods are only for vegetarians and vegans (Connors et al., 2001; de Boer et al., 2017) and question the use of meat-like names (Van Loo et al., 2020). Men have particularly strong meat-eating identities because meat is traditionally associated with masculinity, dominance, and power (Mertens & Oberhoff, 2023). When dining out in groups, men may order a meat dish to express their identity and signal their status, especially to potential romantic partners (Chan & Zlatevska, 2019).

Closely related to food related identities is the social norm of eating meat, a factor which reduces the opportunity to order plant-based meat dishes. Because vegetarians account for around 5% of consumers (IPSOS, 2018), eating meat is a highly visible social norm (Sparkman et al., 2020). Many consumers experience peer pressure to order a meat dish when dining out in a group (Biermann & Rau, 2020; Collier et al., 2021) because they do not want to be stigmatised as a vegetarian (Graça et al., 2019). Common restaurant practices reinforce the social norm of eating meat, including serving meat as the default option, and offering more meat than plant-based options. The cost and perceived value of different menu items is an important factor influencing food choices in restaurants (Ozdemir & Caliskan, 2015). Consumers are willing to pay for meat dishes because they require specific skills, expensive ingredients, and time to create. Plant-based meat dishes are, however, perceived as too expensive because they only contain plants and are easy to prepare (Collier et al., 2021; Kerslake et al., 2022). Plant-based meat dishes are also, on average, more expensive than meat which further reduces the opportunity to order such products (Good Food Institute, 2023). Different factors contribute to the higher cost of plant-based meat. One of the main reasons is that the price of food often does not reflect the true cost of the product. The true cost of food includes the positive and negative impact of the product on human and planetary health, as well as society (Martin-Rios et al., 2023). Adjusting food prices for their environmental footprint would increase the average price of meat products in Germany, for example, by up to €4.42 per kg, while the average price of plant-based products would increase by only up to €0.79 per kg (Michalke et al., 2023).

Overall, consumers who are more open towards plant-based meats tend to be younger (Hoek et al., 2011a), female (Beacom et al., 2021; Götze & Brunner, 2021; Lemken et al., 2019), have a higher education (Hoek et al., 2004; 2011a; Lemken et al., 2019), live in smaller households (Hoek et al., 2004), and reside in urban areas (Beacom et al., 2021; Hoek et al., 2004) compared to consumers who have a strong preference for meat. They tend to be more health conscious, have more knowledge of food and nutrition, care more about the environment (Götze & Brunner, 2021) and animals (Hoek et al., 2011a), think that meat has negative impacts on their own health and the planet (Lemken et al., 2019) and put less emphasis on enjoying food (Niva & Vainio, 2021). The main reasons why consumers do not eat plant-based meats differ between the segments. While meat lovers do not enjoy the taste of plant-based meat, meat avoiders think these products are too expensive, lack the skills to cook them (Knaapila et al., 2022), and they do not like eating unfamiliar foods (Hoek et al., 2011a; Niva & Vainio, 2021). We provide a detailed summary of the segmentation and descriptor variables, data analysis, and results of the existing plant-based meat segmentation studies in Appendix A.

The existing literature provides valuable insights into why consumers are not willing to reduce their meat consumption at home. These findings may not necessarily be transferable to the restaurant context, however, because consumers make different food choices when dining out (Nguyen et al., 2022). Because plant-based meats have only recently emerged as a viable meat alternative, little is known about uptake barriers in general, and in the restaurant context in specific. Initial qualitative studies suggest that the barriers for plant-based meats may well be distinctly different than those preventing people from following a vegetarian or vegan diet, including, for example, that consumers think plant-based meats are unhealthy because of the way they are processed and their high soy content. Also unknown is whether all diners share the same concerns that prevent them from ordering plant-based dishes when eating out, or whether distinct segments of the market exist that have different barrier patterns.

The present study contributes to knowledge in two ways: (1) we conceptualise consumer heterogeneity as differences in patterns of barriers that keep specific consumer groups from ordering plant-based meat dishes, and (2) extend the investigation to the restaurant context, which, to date, has not been covered. Results have immediate practical implications: segmenting consumers based on their consumption barriers and profiling them with restaurant specific variables and consumption behaviour in addition to socio-demographic variables provides tourism researchers and practitioners with valuable insights on how to design target group specific interventions aimed at enticing restaurant patrons to order plant-based meat dishes.

## Research method

### Participants

We conducted a survey study to identify the consumption barriers of the market as a whole as well as consumer segments with distinct plant-based consumption barrier patterns and profile them with restaurant specific and personal characteristics. Segmentation studies typically rely on survey data (Dolnicar et al., 2016) because surveys allow for collecting psychographic, sociodemographic, and behavioural data from a large sample of consumers. We recruited 711 participants from Australia who were at least 18 years old through the online survey panel provided by Prolific Academic. Participants from Prolific Academic provide responses of higher quality compared to other platforms because they are less familiar with academic research questions and provide more genuine answers (Eyal et al., 2021). We removed 64 participants who failed an attention check (requiring them to follow a specific instruction in their response), resulting in a final sample of 647 respondents. Each participant received AUD$2.33 which is in line with Prolific’s compensation guidelines. The University Human Ethics Committee approved this study (2021/HE000110). We conducted the research in Australia because Australia had the fourth highest annual meat consumption per person (115.47 kg) globally in 2019 (Ritchie & Roser, 2019) which is above the recommended daily intake (Nutrition Australia, 2021).

### Questionnaire

The questionnaire consisted of four sections (see full questionnaire in Appendix B). First, we introduced participants to the topic by providing a definition of plant-based meats and showing pictures of products available in Australia. We asked participants to share their opinion on restaurants that offer plant-based meats using an open-ended question to get them thinking about the research context. Participants then indicated how often they order a meat dish, a plant-based meat dish and a vegetarian/vegan dish without plant-based meats when dining out by allocating 100% across the three dish types.

We then encouraged participants to imagine having dinner at a casual dining restaurant that offers plant-based meat dishes. We included a picture of a restaurant to assist participants with their imagination. Next, we showed participants 32 reasons why other people do not order plant-based meat dishes in restaurants in random order and asked them to indicate which reasons prevent them personally from selecting a plant-based meat dish using a binary answer format (agree/disagree). We specifically asked participants to indicate the reasons preventing them from ordering a plant-based meat dish because simply asking participants to reflect on how they perceive plant-based meat would not have been sufficient to understand their plant-based meat consumption barriers. Perceiving foods as unhealthy, for example, does not necessarily prevent consumers, including those who are trying to lose weight, from eating these foods (Verhoeven et al., 2015). We searched the Web of Science and Google Scholar databases using the search terms “barrier OR inhibitor OR motivation” AND “plant-based OR vegetarian OR vegan OR meat alternative OR meat substitute” to identify peer-reviewed articles which determined barriers preventing consumers from eating plant-based meat (e.g., He et al., 2020; Jahn et al., 2021; Kerslake et al., 2022; Onwezen et al., 2021; Szenderák et al., 2022; Weinrich, 2019), and adopting a plant-based (e.g., Fehér et al., 2020; Giacalone et al., 2022; Graça et al., 2019; Hartmann & Siegrist, 2017) or vegetarian diet (e.g., Corrin & Papadopoulos, 2017; Stoll-Kleemann & Schmidt, 2017).

Next, respondents were asked about their restaurant experiences. We modified Götze and Brunner (2021)’s food attribute importance items to fit them to the restaurant context by asking participants to indicate how important it is to them when having dinner at a restaurant to (1) eat a delicious meal, (2) eat a healthy meal, (3) eat an environmentally sustainable meal, (4) eat a meal that’s good value for money, (5) eat foods they usually eat. We presented the items in random order and participants rated each on a slider scale ranging from not important at all (0) to extremely important (100). We used a slider scale to avoid problems associated with ordinal answer formats (Dolnicar, 2013). Participants then indicated how many times a month they had dinner at a restaurant, how much they pay per person on average per restaurant visit, and how often they go to fast food restaurants (counter service, fast delivery, low priced), casual dining restaurants (counter or table service, casual environment, moderately priced), and fine dining restaurants (table service, elegant environment, high priced) by allocating a percentage out of 100%. We adopted pre-existing items from Ponnam and Balaji (2014) to examine motives to visit a restaurant. Specifically, participants indicated how often they go out for dinner to (1) spend time with a partner, (2) celebrate a special occasion, (3) socialise with family and friends, (4) eat a meal without needing to cook. We randomised the order of the motives and used a slider scale ranging from 0% of the time to 100% of the time. Next, participants indicated how often they go out for dinner (1) alone, (2) with their partner (3) with their partner and children, (4) with relatives, (5) with friends, and (6) with work colleagues (Díaz-Méndez & García-Espejo, 2017). We also asked how often participants go out with someone who does not eat meat because having close contact to vegetarians and vegans influences one’s own meat consumption (Hoek et al., 2004; Lemken et al., 2019) using a slider scale ranging from 0% of the time to 100% of the time.

In the third section, participants indicated to what extent they describe themselves as someone who (1) cares about the environment, (2) cares about their fitness, (3) cares about other people’s opinions, (4) cares about their health, (5) loves animals, (6) knows a lot about plant-based meats, (7) knows a lot about nutrition, (8) knows a lot about climate change, (9) is open to new experiences on a slider scale ranging from absolutely not (0) to absolutely (100). Questions were presented in random order. The final section of the questionnaire included socio-demographic questions about gender, age, education, household size, and income. We also asked participants which political party they voted for in the Australian federal election 2022 and if they follow any diet. We pilot tested the survey using a think aloud verbal protocol (Ericsson & Simon, 1998). Ten respondents shared their thoughts while completing the survey to ensure respondents understand and respond to the questions as intended.

### Data analysis

We conducted the analysis at the level of the barrier, rather than creating factors. The reason for this approach is that each barrier is unambiguous and allows clear managerial recommendations to be derived. Creating factors, on the other hand, blurs the clarity of barriers, making it more difficult to derive practical recommendations. In addition, methodologically, exploratory factor analysis assumes population homogeneity, whereas the subsequent segmentation analysis assumes heterogeneity in the population. Factor-cluster analysis, therefore, is not a suitable analysis for revealing or constructing actionable segments (see also Dolnicar & Grün, 2008).

A descriptive analysis provided average agreement rates with each of the 32 barriers across all participants. To account for consumer heterogeneity, we extracted consumer segments by performing block-clustering (also referred to as bi-clustering) using a finite Bernoulli mixture model and assigning respondents to segments based on the estimated maximum posterior probabilities of segment memberships (Singh Bhatia et al., 2017). In the block-clustering model, segments of respondents consistently show similar agreement levels for groups of barrier questions, i.e., respondents and questions are simultaneously clustered. For each combination of respondent segment and question group, the agreement level is assumed to be the same and given respondent segment and question group membership, the responses are independently drawn from a Bernoulli distribution. A block-clustering approach simultaneously groups respondents and questions and, in so doing, creates a more parsimonious model compared to a model that only clusters respondents. The block-clustering approach assumes that the agreement levels to each barrier question are identical across question groups within each of the respondent segments. This approach requires, therefore, only as many parameters for each respondent segment as there are question groups. Reducing the number of parameters per respondent segment and regularising the agreement levels across question groups facilitates extracting a higher number of meaningful respondent segments.

The block-clustering model is estimated for a fixed number of respondent segments and groups of barrier questions using maximum likelihood estimation with the block expectation maximisation algorithm as proposed in Govaert and Nadif (2008). The number of respondent segments and groups of barrier questions are varied between 1 and 10 and the best solution is selected based on the integrated completed likelihood criterion (Biernacki et al., 2000). Using the block-clustering model, a segmentation of the respondents is determined based on their final posterior probabilities of segment memberships. Based on this respondent partition, the segments are characterised with respect to the barrier questions based on the segment specific average agreement levels. This implies that characterisation of the segments is based on the partition obtained and not the fitted block-clustering model. Questions are grouped to reduce the complexity of the model and enable the estimation of a more parsimonious model, thus avoiding overfitting heterogeneity in the questions and enabling the extraction of more respondent segments. Our analysis focuses on the specific questions and not on constructs derived from aggregating responses across several questions. We, therefore, do not provide a detailed report on the estimated question groups but interpret the results at the level of the barrier.

Relevant barriers to plant-based meat consumption are directly identified by respondents and focus is given to extract groups of respondents with similar barrier patterns. We use restaurant specific variables to profile the segments, including consumption frequency of plant-based meat dishes. Profiling of the segments with respect to the additional background variables is performed by comparing their segment-specific distributions across segments. Association between the additional background variables and the segment memberships is assessed based on an analysis of variance for metric background variables (e.g., age, number of restaurant visits) and Fisher’s exact test for categorical and binary background variables (e.g., diet). The alternative approach of identifying a suitable regression to predict plant-based meat consumption based on the answers to the barrier questions is unfeasible due to the observational nature of the data. We performed the analysis in the R environment for statistical computing and graphics (Version 4.3.1) (The R Foundation, 2023) using particularly the contributed package blockcluster (Iovleff & Singh Bhatia, 2023) for fitting the block-clustering model.

## Results

### Descriptive results

The final sample included 50% women and the average age was 34.6 years (standard deviation = 12.3 years) old. Thirty-one percent of participants had completed a tertiary education and the average household size was three people. Compared to the Australian population, the sample of this study is representative in terms of gender distribution and household size, but is, on average, slightly younger (the median age of Australian population is 38 years) and better educated (28% of Australians above 18 have completed a tertiary education) (Australian Bureau of Statistics, 2022a, 2022b). These deviations from the general population reflect the typical Australian restaurant patron who tends to be younger and better educated (Cameron et al., 2022; Hogan, 2018). Participants in this study eat dinner at a restaurant 3.6 times per month, on average, and spend AUD$37.11 per visit. Socialising with family and friends (on average 67% of the time), eating a meal without needing to cook (56), celebrating a special occasion (55), and spending time with a partner (52) are the main reasons for having dinner at a restaurant. Participants most frequently dine with a partner (54), friends (50), relatives (41) or alone (17). When deciding which dish to order, the most important factors are taste (92) and price (78). On average, participants order a meat dish 69%, a vegetarian/vegan dish without plant-based meat 24%, and a plant-based meat dish 7% of the time when dining out. Seventy-eight percent do not follow any specific diet, and 68% voted for a progressive party. Overall, participants care about the environment (74), their health (74) and fitness (65) and are open to new experiences (74). Participants have limited knowledge of plant-based meats (37), nutrition (57) and climate change (61). Appendix C provides a detailed overview of the background variables.

**Figure 2.**
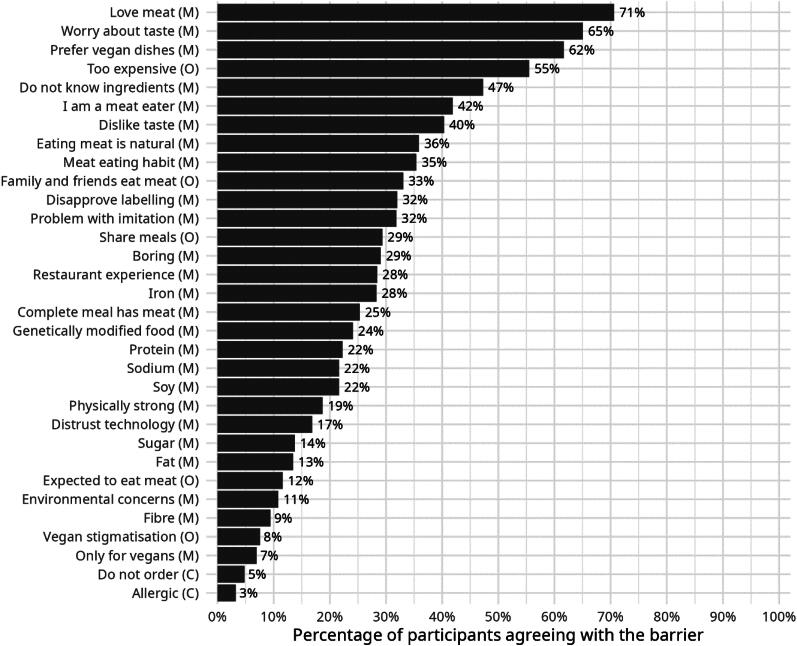
Percentage of participants agreeing with the barrier. *Note.* Capability (C), Opportunity (O), Motivation (M).

Figure 2 shows the barriers for the population of people who dine out as a whole, at aggregate level. Although this is not the primary aim of the paper, it is still an interesting descriptive result worth reporting because it paints an overarching picture of barriers perceived by consumers. As can be seen, the main barriers to ordering a plant-based meat dish relate to motivation. Specifically, consumers do not order plant-based meat dishes because they love the taste of meat (71%), worry about not enjoying plant-based meats (65%), prefer vegan/vegetarian dishes without plant-based meats (62%), and do not enjoy the taste of plant-based meat dishes (40%). Not knowing what plant-based meats are made of (47%) and having a meat-eating identity (42%) are additional factors which reduce participants’ motivation to order a plant-based meat dish. The price of plant-based meats is a common factor that reduces participants’ opportunity to order plant-based meat dishes (55%). Less than 10% of participants said they would not order plant-based meat dishes because of allergies (3%), someone else orders (5%), plant-based meats are for vegans only (7%), fear of being stigmatised as a vegetarian and vegan (8%) and not enough fibre (9%). Between 10% and 40% of participants mentioned all remaining barriers.

### Segmentation

**Figure 3.**
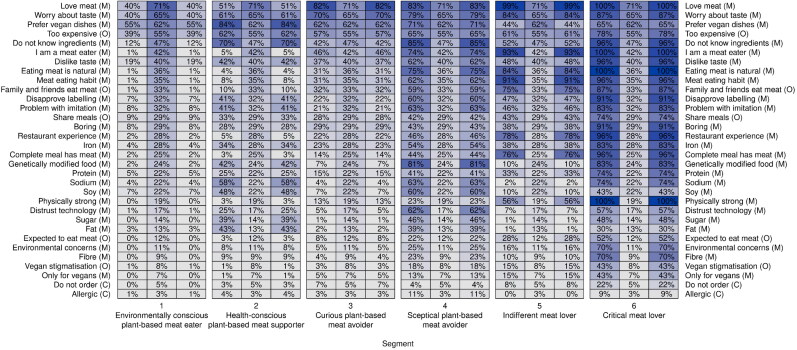
Segmentation of restaurant guests based on the reasons for not ordering plant-based meat dishes. *Note.* Capability (C), Opportunity (O), Motivation (M).

The block-clustering model created six respondent segments as well as eight barrier question groups when using the integrated completed likelihood criterion to select a suitable model. Respondents were segmented by assigning them to the segment where their a-posteriori probability was maximum. We ordered the segments from having small to high numbers of plant-based meat consumption barriers and labelled them as follows: environmentally conscious plant-based meat eater (segment 1, 25.5% of participants), health-conscious plant-based meat supporter (segment 2, 12.2%), curious plant-based meat avoider (segment 3, 30.6%), sceptical plant-based meat avoider (segment 4, 14.7%), indifferent meat lover (segment 5, 13.4%), critical meat lover (segment 6, 3.6%). The segmentation solution is visualised using a block-clustering plot in Figure 3 (Dolnicar et al., 2012). In this plot, columns represent segments and rows barriers. The column for each segment is split into three parts where the left and right part indicate the segment-specific agreement level with the barrier and the middle part the population agreement level, thus allowing to contrast the segment with the general population. This visualisation emphasises the segment-specific values which are included twice in the left and right part while still allowing a comparison with the aggregate values. The colours of the cells range from white to blue with increasing level of agreement and the percentages of agreement are also inserted in the cells to facilitate the comparison.

#### Environmentally conscious plant-based meat eater (segment 1)

The environmentally conscious plant-based meat eater scored below average on each barrier. The only two emerging motivational barriers are their preference for vegetarian and vegan dishes without plant-based meat (55%) and worrying about not enjoying plant-based meat dishes (40%). A participant described their hesitancy towards ordering a plant-based meat dish as follows “*I don’t have any issues with plant-based meats and eat them at home occasionally, but I tend not to order plant-based meat options when at a restaurant. I prefer to know which brands/types of plant-based meats are being used as there are certain ones I like and others that are too similar to animal meat. When ordering at a restaurant that I haven’t been to before I tend to pick the “safe” vegetarian options.”* Overall, this segment supports restaurants that offer plant-based meat dishes and describes them as “*inclusive*” and “*ethical*”.

Compared to the other segments, the environmentally conscious plant-based meat supporter orders more vegetarian dishes with (on average 15% of the times) or without plant-based meat (37) when dining out. This segment has the greatest share of vegetarians/vegans (30%) and dines most frequently with a person who does not eat meat (39). When making food choices in restaurants, the typical consumer in this segment considers the environmental footprint of the dish (53) and looks less for healthy foods (54) and foods they always eat (39). The typical consumer in this segment cares deeply about the environment (78) and is knowledgeable about climate change (66) and plant-based meats (47). They believe strongly that eating less meat is required to mitigate climate change (73). However, they care less about their fitness (62) and health (72). This segment has on average the youngest consumers (32 years), has the greatest share of women (60%) but the lowest number of participants who completed a tertiary education (63%). Almost all environmentally conscious plant-based meat eaters (99%) voted for a progressive party (e.g., labour or greens) in the 2022 Australian federal election.

#### Health-conscious plant-based meat supporter (segment 2)

The main factors reducing the motivation of the health-conscious plant-based meat supporter to order plant-based meat dishes relate to the taste and healthiness of these products. Taste barriers include preferring vegan/vegetarian dishes without plant-based meats (84%), worrying about not enjoying plant-based meat dishes (62%), and not liking the taste of plant-based meats (62%) as evidenced by the following quote: *“while I’m generally in favour of them, I think that using plant-based meats often leads to an inferior dish. It’s perfectly possible to make delicious vegetarian or vegan food without fake meat.”* Consumers in this segment believe plant-based meats are unhealthy because they contain too much sodium (58%), soy (48%), fat (43%), genetically modified ingredients (42%), and sugar (39%). One participant described plant-based meat as “*very unappetizing, do not have any interests in this kind of heavily processed food and high sodium food. I’d rather just eat the vegetables they used to make the fake meat”.* Unfamiliarity with these products (70%) and the apprehension towards mimicking meat with plants (41%) and using animal-meat like names to describe them (41%) also reduce the motivation to order such dishes as evidenced by the following quote: “*I think it’s important for customers to know what is in a plant-based meat, and for the name of the dish to reflect that. If I want to order a bean, potato, lentil type schnitzel, for example, then it’s very unappealing to have it called ‘vegetarian beef schnitty’—call it what it is, I want to be confident in what I’m ordering.”* The perception that plant-based meat dishes are too expensive (62%) is the predominant factor limiting the opportunity to order these products for consumers in this segment. Overall, this segment is supportive of restaurants offering plant-based meats but would like to have more information about the products, particularly about the ingredients.

Like the environmentally conscious plant-based meat eater, consumers in this segment order a vegetarian/vegan dish without (on average 42% of the times) or with plant-based meat (8) more often compared to the other segments. This segment has the second highest number of vegetarians/vegans (18%) and frequently dines with someone who does not eat meat (37). When deciding what dish to order, this segment looks for healthy (67) and environmentally sustainable dishes (60) and does not prioritise eating foods they always eat (42).

The typical health-conscious plant-based meat supporter cares about their fitness (74), health (81) and the environment (78) and is knowledgeable about nutrition (65) and plant-based meat (43) and believes strongly that eating less meat is necessary for mitigating climate change (69). This segment is older (37 years), has the second highest share of women (58), and is well educated with 80% of participants having completed a tertiary education.

#### Curious plant-based meat avoiders (segment 3)

The typical curious plant-based meat avoider lacks the motivation to order plant-based meat dishes because they prefer meat (82%) or traditional vegetarian/vegan dishes without plant-based meat (62%). Consumers in this segment are also unfamiliar with plant-based meat (42%) and worry they will not enjoy the taste of these dishes (70%): “*I think if I were offered a sample, I would be more inclined to try it but I’m already very picky with my choices and order what I know I will like—the risk of it being disappointing does not justify the cost to me”.* The price of plant-based meat (62%) is the dominant factor reducing the opportunity to order plant-based meat dishes because it “*feels lazy and not worth paying for. I would eat it if it was cheaper. The pre-made factor of plant-based meals makes it feel like I could just buy the meat and make it at home.”* Overall, this segment is supportive of restaurants offering plant-based meat dishes and would consider ordering them if they were priced similar to meat and they knew they would enjoy it.

This is the first segment that orders more meat dishes (on average 77% of the times) than vegetarian dishes. The typical consumer in this segment does not look for foods they normally eat (44) and considers less the environmental footprint (50) or nutritional value of the dish (56). Only 2.5% of this segment follow a vegan/vegetarian diet, but they dine on average 28% of the time with someone who does not eat meat. The curious plant-based meat avoider believes that eating less meat is somewhat important for mitigating climate change (63). The typical consumer in this segment cares less about their fitness (61) and health (72) but more about what others think (58). They are not knowledgeable about plant-based meats (33) or nutrition (53). This segment consists of younger consumers (32 years) with an even distribution of men and women, and almost exclusively people who voted progressively (92%).

#### Sceptical plant-based meat avoider (segment 4)

Consumers in this segment are not motivated to order plant-based meat dishes because they prefer meat dishes (83%) or traditional vegetable dishes (71%), are unfamiliar with plant-based meat dishes (85%) and worry they will not enjoy the taste of such dishes (79%) as evidenced by the following quote: “*I love meat dishes and vegetable dishes and eat vegetables with most meals. If I am going to eat a meat dish I would like real meat. Food replacements are … no substitute for the real thing and compromise on taste.”*

Health concerns are also present among the sceptical plant-based meat avoider because they believe plant-based meats contain too many genetically modified ingredients (81%), too much soy (60%), sodium (63%), sugar (46%), fat (39%), and not enough iron (54%) and protein (41%) as highlighted by this quote: “*I am very concerned about what is used to make plant-based meats…. Reading the back of plant-based meat packages will typically reveal a plethora of chemicals. I’d rather have animal meat where I know exactly what the singular ingredient is: meat.”*

This segment that distrusts the technology used to create plant-based meat (62%) nor supports the idea of mimicking meat with plants (63%) and labelling these products with animal-meat like names (60%): “*If they didn’t use the plant-based meat terminology I would be inclined to eat it.”* Meat eating habits (62%) and identifying as a meat eater (72%) further impact the motivation to select a plant-based meat dish. The price of plant-based meat (65%) and the meat ordering behaviour of their peers (59%) reduce the opportunity to order plant-based meat dishes for the sceptical plant-based meat avoider. Sharing meals with others (42%) reduces the capability to order plant-based meat dishes. Overall, this segment appreciates that restaurants use these plant-based meats to cater to people who do not want to eat meat, but themselves prefer eating traditional meat or vegetable dishes.

This segment orders most frequently a meat dish (on average 78% of the times) and occasionally a vegetarian/vegan dish without plant-based meats (17). When deciding what to order, the typical consumer in this segment looks for heathy dishes (66), foods they usually eat (58) and considers the environmental footprint of the meal to a lesser extent (47). They care less about the environment (70), know less about climate change (56) and plant-based meats (21) and do not think eating less meat is important for mitigating climate change (42). This segment is on average 40 years old, has an even distribution between men and women and 77% voted for a progressive party.

#### Indifferent meat lover (segment 5)

The typical indifferent meat lover lacks motivation to order plant-based meat dishes because they identify as meat eaters (93%), love meat (99%), order meat dishes out of habit (91%), and believe eating a meat dish is part of the dining experience (78%) as evidenced by the following quote: “*I wouldn’t know how you’d mimic meat sliding off a bone because it has been cooked so perfectly. If I’m going out to a restaurant and ordering food, I don’t see myself ever ordering pasta with a Bolognese sauce that’s made with plant-based meat when I can just have standard meat.”* Worring of not enjoying the taste of plant-based meat (84%) also reduces the motivation “*something about the effort and price of going to a restaurant makes me want to stick with something I know I will enjoy (i.e., animal meat meals).”*

While consumers in this segment do not have any particular health concerns regarding plant-based meat, they believe eating meat is natural (84%), necessary to feel physically strong (56%) and that a complete meal contains meat (76%): “*I think there is a nutritional requirement for animal meat inherent in humans so plant-based meat in the long term might serve more of a complementary role rather than a supplementary one when it comes to animal meat.”* Almost half of all consumers in this segment (46%) do not support the idea of mimicking meat using plants and labelling them with animal-like names (47%): “*I think it’s dishonest to label them as meat alternatives or try to name them similar to the actual meat product. Label them as what they are and stop trying to pretend they’re something they’re not.”* The indifferent meat lover has a limited opportunity to order plant-based meat dishes because of the price of such dishes (61%) and the meat ordering behaviour of family and friends (75%). The consumer in this segment only occasionally dines with someone who does not eat meat (17%) and almost every second consumer in this segment does not order plant-based meat dishes because they share meals with people who do not want to eat plant-based meats (43%) which reduces the opportunity to order such dishes.

Overall, this segment supports restaurants that offer plant-based meat dishes because they cater for guests who do not want to eat meat. However, they would not consider ordering a plant-based meat dish and believe that offering plant-based meats should not reduce the number of available meat dishes. Labels should clearly differentiate between meat and plant-based meat dishes to avoid any misunderstandings.

When dining out this segment eats predominantly meat dishes (on average 92% of the times), foods they usually eat (60). The typical consumer in this segment cares less about the environment (65) and is less knowledgeable about plant-based meat (26), climate change (54) and nutrition (53). The indifferent meat lover does not believe eating less meat is required to mitigate climate change (42). Consumers in this segment are on average 36 years old and are mostly male (71%) with nearly 74% having voted for a progressive party.

#### Critical meat lover (segment 6)

Like segment 5, the typical critical meat lover is not motivated to order plant-based meat dishes because they love the taste of meat (100%) and dislike plant-based meats (96%). They do not know what plant-based meats are made of (96%) and worry they will not enjoy them (87%). They always eat meat when dining out (96%) and believe that meat is an important part of the restaurant experience (96%). All consumers in this segment believe they need meat to feel physically strong and think that plant-based meats are unhealthy, particularly, because of their low amount of iron (83%), protein (74%), fibre (70%) and too high amounts of genetically modified ingredients (83%) and sodium (74%). Critical meat lovers identify as meat eaters (100%) and believe plant-based meats are only for vegetarian/vegans (43%) and do not want to be stigmatised as someone who does not eat meat (43%). Among all segments, only critical meat lovers think plant-based meats are environmentally harmful (70%) and that eating less meat is important for mitigating climate change (39%). They also do not trust the technology used to create these dishes (57%) and do not support mimicking meat with plants (83%) and labelling them with animal-like names (91%) as evidenced by the following quote: “*occasionally these have been sold under misleading pretence. Several times I have eaten this garbage without knowing what I was ordering and thoroughly regretted it. I really wish this garbage didn’t exist.”*

Almost all their family and friends eat meat (87%) and expect them to eat meat (52%) which reduces the opportunity to order plant-based meat dishes. Overall, the critical meat lover does not see the need for restaurants to offer plant-based meat dishes. Seventy-four percent of respondents in this segment indicated they do not have the opportunity to order plant-based meat dishes because they share meals with others who do not want to eat such dishes. Like segment 5, this segment orders almost always meat dishes when dining out (on average 90% of the times) and chooses healthy foods (72) they normally eat (74). The typical critical meat lover cares less about the environment (43) and is not knowledgeable about plant-based meats (32). The average critical meat lover is 37 years old and most consumers in this segment are male (78%) and voted for a conservative party (60%).

## Discussion

This study makes three key contributions to the existing literature on plant-based meat consumption: (1) it uses the COM-B model as theoretical foundation to test which barriers are most dominant for restaurant guests, (2) it identifies heterogeneity in the barriers acknowledged across consumer groups, and (3) it uses variables relevant to the restaurant context to profile consumer groups, including previous consumption of plant-based meat dishes.

Our study identifies taste preferences for meat and traditional vegetable dishes along with the worry of not enjoying a plant-based meat dish and health concerns as main factors reducing the motivation for ordering a plant-based meat dish. This study extends prior research that suggests consumers believe plant-based meat is unhealthy by identifying the main ingredients that create this perception: sodium, soy, protein, iron, and genetically modified ingredients. The price of plant-based meat emerges as the main factor that reduces consumer opportunity to order a plant-based meat dish, 55% of the sample indicated price as a barrier, which confirms findings from the home context (Gómez-Luciano et al., 2019). This study contributes to the existing literature on heterogeneity between consumer groups in plant-based meat consumption at home (Götze & Brunner, 2021; Knaapila et al., 2022) by identifying six distinct segments based on uptake barriers in restaurants. The environmentally conscious plant-based meat eater (segment 1) and the health-conscious plant-based meat supporter (segment 2) occasionally order plant-based meat dishes and frequently order traditional vegetable dishes when dining out. While segment 1 does not have any dominant reasons for not ordering plant-based meats, segment 2 believes they are not healthy. The curious plant-based meat avoider (segment 3) and the sceptical plant-based meat avoider (segment 4) are flexitarians who are considering ordering plant-based meat dishes. Both segments worry they will not enjoy plant-based meat dishes and segment 4 also believes these products are unhealthy. The indifferent meat lover (segment 5) and the critical meat lover (segment 6) are meat lovers who will not order a plant-based meat dish. While consumers in segment 5 simply love eating meat and believe it is a critical component to the restaurant experience, segment 6 also strongly disagrees with the concept of using plant-based foods to imitate meat products.

Based on the findings of this study, we propose several segment-specific interventions to entice consumers belonging to segments 1 to 4 to order a plant-based meat dish as shown in Table 1. We focus on these segments because segments 5 and 6 are unlikely to change their ordering behaviour. Menu design approaches have shown promising results in altering food choices in restaurants (e.g., Demeter et al., 2023; Greene et al., 2023). The COM-B model proposes education, persuasion, incentives, and environmental restructuring interventions (Michie et al., 2011). Education interventions aim to increase knowledge and understanding of plant-based meats. Segments 2 and 4 express concerns about the healthiness of plant-based meats. Providing these consumer groups with information about the nutritional value of different menu items represents a promising approach (Cozzio et al., 2022). Segments 1 and 2 care deeply about the environment and consider the environmental footprint of menu items when making food choices. Promoting the environmental benefits of plant-based meats may, therefore, encourage these consumer groups to select a plant-based meat dish (Malan et al., 2022; Ye & Mattila, 2021, 2022). Future research could test additional education-based interventions, such as informing restaurant patrons of the manufacturing process and ingredients of plant-based meats.

**Table 1.** Segment-specific interventions based on the COM-B model.

Intervention	Consumption barrier	Segment 1	Segment 2	Segment 3	Segment 4
**Education interventions**					
Provide health information	Health (M)		**✓**		**✓**
Provide environmental information	Environment (M)	**✓**	**✓**		
Provide information about production process	Health (M) and familiarity (M)		**✓**	**✓**	**✓**
Provide information about ingredients	Familiarity (M)	**✓**	**✓**	**✓**	**✓**
**Persuasive interventions**					
Use indulgent language	Taste (M)		**✓**	**✓**	**✓**
Use appealing images	Taste (M)		**✓**	**✓**	**✓**
Offer free samples	Taste (M)		**✓**	**✓**	**✓**
Avoid unappealing language	Identity (M)				**✓**
Use different labels	Identity (M) and imitation (M)		**✓**		**✓**
Use social norm messaging	Social norm (O)				**✓**
**Incentives**					
Reduce price of plant-based meat dishes	Price (O)		**✓**		**✓**
Offer rewards for plant-based meat dishes	Price (O)		**✓**		**✓**
Run cross-product promotions on plant-based meat dishes and selected drinks, side dishes, or desserts	Price (O)		**✓**		**✓**
Run multibuy or buy-one-get-one-free offers	Price (O)		**✓**		**✓**
Give diners coupons or loyalty card points to redeem on plant-based meat dishes	Price (O)		**✓**		**✓**
**Environmental restructuring**					
Increase number of plant-based meat dishes	Social norm (O)				**✓**
Serve plant-based meat dishes as the default option	Social norm (O) and habit (M)				**✓**
Place plant-based meat dishes in more visible locations	Habit (M)				**✓**
Integrate plant-based meat dishes in full menu	Habit (M) and identity (M)				**✓**

*Note:* Capability (C), Opportunity (O), Motivation (M).

Persuasion interventions aim to create more positive feelings and attitudes towards plant-based meats among restaurant guests. Segments 2 to 4 dislike the taste of plant-based meat or worry they will not enjoy these dishes. Considering that segments 2 to 4 worry about the taste of plant-based meat, determining if describing these dishes with indulgent words (Greene et al., 2023), using appealing images of the dishes (Fechner et al., 2023), or providing free samples affects ordering behaviour is an important future research area.

Segment 4 thinks of themselves as meat eaters and believes eating meat is normal. Avoiding words which may create the perception that plant-based meats are not intended for meat eaters, such as meat-free, vegan, or vegetarian (Bacon et al., 2018) and using messages highlighting that a growing number of restaurant guests has recently selected plant-based meat dishes present promising avenues to entice these consumers to order a plant-based meat dish (Sparkman et al., 2020). Because segments 2 and 4 disapprove of labelling products made from plants with animal-meat like names, testing different labelling strategies represents an important future research area.

Incentives provide restaurant guests with a financial reward for selecting a plant-based meat dish. Offering plant-based meat dishes at a discounted price presents a promising incentive intervention (Garnett et al., 2021), especially for consumers belonging to segments 2 to 4. Future research could test cross-product promotions on plant-based meat dishes and other menu items, multi-buy or buy-one-get-one-free offers, or the provision of coupons and extra loyalty points for plant-based meat dishes (World Resources Institute, 2020).

Environmental restructuring includes changes to the physical and social context in which restaurant guests make food choices. Changes to the environment help draw attention to plant-based meat dishes and normalise eating them. Increasing the number of available plant-based meat dishes (Malan et al., 2022), changing the default to serving plant-based meat dishes (Taufik et al., 2022), placing plant-based meat dishes at the top of the menu (Andersson & Nelander, 2021) and in more visible locations on counters (Garnett et al., 2020) create an environment that entices consumers to order plant-based meat dishes and may disrupt the meat ordering habit. Furthermore, integrating plant-based meat dishes in the full menu rather than listing them in a separate vegetarian section can encourage consumers who think of themselves as meat eaters (segment 4) to consider ordering these dishes (Vandenbroele et al., 2021).

This study has a few limitations. First, it focuses on plant-based meats, limiting the generalisability of the findings to other alternative proteins such as cultivated meat and more traditional meat alternatives including tofu and tempeh. Future research could extend this study by comparing the identified segments in this study to other alternative protein types. Examining additional descriptor variables, such as intention towards behaviour change, could also provide valuable insights into how to design interventions. Second, we collected data from consumers in Australia where plant-based meats are an emerging food and eating high amounts of meat is part of the culture and a social norm. The generalisability of the findings to countries with a long history of plant-based meats and a more plant-dominant diet such as certain Asian countries (Ortega et al., 2022) might be limited. Third, participants imagined having dinner at a casual dining restaurant. Using fast food or fine dining restaurants as research context may lead to different segments because dining motives may change when imagining having dinner at these restaurant types (Xu & Jeong, 2019). Consumers may also respond differently to plant-based meat in other contexts, such as workplaces, schools, universities, or hospitals. Fourth, while we extracted the segments based on distinct plant-based meat consumption barrier patterns and profiled each segment using their plant-based meat consumption frequency, field experiments are needed to test which of the recommended segment-specific interventions are most effective in enticing consumers to order a plant-based meat dish. All of these limitations present opportunities for future work in this area, as does the systematic investigation of facilitators (as opposed to barriers) of plant-based meat consumption. While barriers have to be overcome with interventions, facilitators can be leveraged, thus offering a valuable alternative direction for increasing demand.

## Conclusion

The tourism sector can play an important role in creating a food system in which environmentally sustainable, healthy, delicious, and culturally appropriate dishes are the convenient, affordable, and socially acceptable choice while securing livelihoods and preserving the cultural heritage of food. The transition to plant-dominant restaurant menus is, however, complex and requires the collaboration and support from different stakeholder groups, including consumers. Informed by the COM-B model, this study identified six consumer segments with distinct plant-based meat consumption barrier patterns. Based on these insights, we provide theoretically informed, target group specific interventions to entice consumers to order plant-based meat dishes, and in so doing, reduce their food related greenhouse gas emissions and support the transition to more environmentally sustainable food systems. Interventions, such as using more indulgent language to describe plant-based meat dishes, can be immediately implemented by restaurants. Other interventions, particularly incentives which rely on selling plant-based meat at a discounted price, may first require political initiatives, such as meat taxes or plant-based meat subsidies. We encourage a close collaboration between the tourism industry, food manufactures, farmers, governments, and indigenous groups to identify opportunities to leverage the tourism sector as a facilitator towards more sustainable food systems.

## Supplementary Material

Supplemental Material

## References

[CIT0001] Andersson, O., & Nelander, L. (2021). Nudge the lunch: A field experiment testing menu-primacy effects on lunch choices. *Games*, *12*(1), 2. 10.3390/g12010002

[CIT0002] Australian Bureau of Statistics. (2022a). Location: Census. Retrieved May 15, 2023, from https://www.abs.gov.au/statistics/people/people-and-communities/location-census/latest-release

[CIT0003] Australian Bureau of Statistics. (2022b). Snapshot of Australia. Retrieved May 15, 2023, from https://www.abs.gov.au/statistics/people/people-and-communities/snapshot-australia/2021

[CIT0004] Bacon, L., Wise, J., Attwood, S., & Vennard, D. (2018). The language of sustainable diets: A field study exploring the impact of renaming vegetarian dishes on UK cafe menus. Retrieved May 12, 2023, from https://wriorg.s3.amazonaws.com/s3fspublic/language-sustainable-diets.pdf.

[CIT0005] Beacom, E., Bogue, J., & Repar, L. (2021). Market-oriented development of plant-based food and beverage products: A usage segmentation approach. *Journal of Food Products Marketing*, *27*(4), 204–222. 10.1080/10454446.2021.1955799

[CIT0006] Benningstad, N. C., & Kunst, J. R. (2020). Dissociating meat from its animal origins: A systematic literature review. *Appetite*, *147*, 104554. 10.1016/j.appet.2019.104554 31830517

[CIT0007] Bhatia, P. S., Iovleff, S., & Govaert, G. (2017). blockcluster: An R package for model-based co-clustering. *Journal of Statistical Software*, *76*(9), 1–24. 10.18637/jss.v076.i0936568334

[CIT0100] Biermann, G., & Rau, H. (2020). The meaning of meat:(Un) sustainable eating practices at home and out of home. *Appetite*, *153*, 104730. 10.1016/j.appet.2020.104730 32417300

[CIT0008] Biernacki, C., Celeux, G., & Govaert, G. (2000). Assessing a mixture model for clustering with the integrated completed likelihood. *IEEE Transactions on Pattern Analysis and Machine Intelligence*, *22*(7), 719–725. 10.1109/34.865189

[CIT0009] Breslin, P. A. (2013). An evolutionary perspective on food and human taste. *Current Biology: CB*, *23*(9), R409–R418. 10.1016/j.cub.2013.04.010 23660364 PMC3680351

[CIT0010] Brooker, P. G., Hendrie, G. A., Anastasiou, K., Woodhouse, R., Pham, T., & Colgrave, M. L. (2022). Marketing strategies used for alternative protein products sold in Australian supermarkets in 2014, 2017, and 2021. *Frontiers in Nutrition*, *9*, 1087194. 10.3389/fnut.2022.1087194 36618675 PMC9815776

[CIT0011] Bryant, C. J. (2019). We can’t keep meating like this: Attitudes towards vegetarian and vegan diets in the United Kingdom. *Sustainability*, *11*(23), 6844. 10.3390/su11236844

[CIT0012] Bryant, C. J. (2022). Plant-based animal product alternatives are healthier and more environmentally sustainable than animal products. *Future Foods*, *6*, 100174. 10.1016/j.fufo.2022.100174

[CIT0013] Bunge, A. C., Wood, A., Halloran, A., & Gordon, L. J. (2022). A systematic scoping review of the sustainability of vertical farming, plant-based alternatives, food delivery services and blockchain in food systems. *Nature Food*, *3*(11), 933–941. 10.1038/s43016-022-00622-8 37118205

[CIT0014] Cameron, A. J., Oostenbach, L. H., Dean, S., Robinson, E., White, C. M., Vanderlee, L., Hammond, D., & Sacks, G. (2022). Consumption frequency and purchase locations of foods prepared outside the home in Australia: 2018 international food policy study. *The Journal of Nutrition*, *152*(Suppl 1), 76S–84S. 10.1093/jn/nxab437 35274693 PMC9188859

[CIT0015] Chan, E. Y., & Zlatevska, N. (2019). Is meat sexy? Meat preference as a function of the sexual motivation system. *Food Quality and Preference*, *74*, 78–87. 10.1016/j.foodqual.2019.01.008

[CIT0016] Clark, L. F., & Bogdan, A.-M. (2019). Plant-based foods in Canada: Information, trust and closing the commercialization gap. *British Food Journal*, *121*(10), 2535–2550. 10.1108/BFJ-12-2018-0826

[CIT0017] Clark, M. A., Domingo, N. G., Colgan, K., Thakrar, S. K., Tilman, D., Lynch, J., Azevedo, I. L., & Hill, J. D. (2020). Global food system emissions could preclude achieving the 1.5° and 2° C climate change targets. *Science (New York, NY)*, *370*(6517), 705–708. 10.1126/science.aba7357 33154139

[CIT0018] Collier, E. S., Oberrauter, L.-M., Normann, A., Norman, C., Svensson, M., Niimi, J., & Bergman, P. (2021). Identifying barriers to decreasing meat consumption and increasing acceptance of meat substitutes among Swedish consumers. *Appetite*, *167*, 105643. 10.1016/j.appet.2021.105643 34389377

[CIT0019] Connors, M., Bisogni, C. A., Sobal, J., & Devine, C. M. (2001). Managing values in personal food systems. *Appetite*, *36*(3), 189–200. 10.1006/appe.2001.0400 11358343

[CIT0020] Corrin, T., & Papadopoulos, A. (2017). Understanding the attitudes and perceptions of vegetarian and plant-based diets to shape future health promotion programs. *Appetite*, *109*, 40–47. 10.1016/j.appet.2016.11.018 27871943

[CIT0021] Cozzio, C., Volgger, M., & Taplin, R. (2022). Point-of-consumption interventions to promote virtuous food choices of tourists with self-benefit or other-benefit appeals: A randomised field experiment. *Journal of Sustainable Tourism*, *30*(6), 1301–1319. 10.1080/09669582.2021.1932936

[CIT0022] de Boer, J., Schösler, H., & Aiking, H. (2017). Towards a reduced meat diet: Mindset and motivation of young vegetarians, low, medium and high meat-eaters. *Appetite*, *113*, 387–397. 10.1016/j.appet.2017.03.007 28300608

[CIT0023] Dembedza, V. P., Chopera, P., Mapara, J., & Macheka, L. (2022). Impact of climate change-induced natural disasters on intangible cultural heritage related to food: A review. *Journal of Ethnic Foods*, *9*(1), 32. 10.1186/s42779-022-00147-2

[CIT0024] Demeter, C., Fechner, D., & Dolnicar, S. (2023). Progress in field experimentation for environmentally sustainable tourism–A knowledge map and research agenda. *Tourism Management*, *94*, 104633. 10.1016/j.tourman.2022.104633

[CIT0025] Díaz-Méndez, C., & García-Espejo, I. (2017). Eating out in Spain: Motivations, sociability and consumer contexts. *Appetite*, *119*, 14–22. 10.1016/j.appet.2017.03.047 28377045

[CIT0026] Dolnicar, S. (2013). Asking good survey questions. *Journal of Travel Research*, *52*(5), 551–574. 10.1177/0047287513479842

[CIT0027] Dolnicar, S., & Grün, B. (2008). Challenging “factor-cluster segmentation. *Journal of Travel Research*, *47*(1), 63–71. 10.1177/0047287508318910

[CIT0028] Dolnicar, S., Grün, B., & Leisch, F. (2016). Increasing sample size compensates for data problems in segmentation studies. *Journal of Business Research*, *69*(2), 992–999. 10.1016/j.jbusres.2015.09.004

[CIT0029] Dolnicar, S., Kaiser, S., Lazarevski, K., & Leisch, F. (2012). Biclustering: Overcoming data dimensionality problems in market segmentation. *Journal of Travel Research*, *51*(1), 41–49. 10.1177/0047287510394192

[CIT0030] Elzerman, J. E., Van Boekel, M. A., & Luning, P. A. (2013). Exploring meat substitutes: Consumer experiences and contextual factors. *British Food Journal*, *115*(5), 700–710. 10.1108/00070701311331490

[CIT0031] Ericsson, K. A., & Simon, H. A. (1998). How to study thinking in everyday life: Contrasting think-aloud protocols with descriptions and explanations of thinking. *Mind, Culture, and Activity*, *5*(3), 178–186. 10.1207/s15327884mca0503_3

[CIT0032] Eshel, G., Stainier, P., Shepon, A., & Swaminathan, A. (2019). Environmentally optimal, nutritionally sound, protein and energy conserving plant based alternatives to US meat. *Scientific Reports*, *9*(1), 10345. 10.1038/s41598-019-46590-1 31395893 PMC6687707

[CIT0033] Eyal, P., David, R., Andrew, G., Zak, E., & Ekaterina, D. (2021). Data quality of platforms and panels for online behavioral research. *Behavior Research Methods*, *54*(4), 1643–1662. 10.3758/s13428-021-01694-3 34590289 PMC8480459

[CIT0034] Fechner, D., Karl, M., Grün, B., & Dolnicar, S. (2023). How can restaurants entice patrons to order environmentally sustainable dishes? Testing new approaches based on hedonic psychology and affective forecasting theory. *Journal of Sustainable Tourism*, 1–20. 10.1080/09669582.2023.2274283 PMC11451025 39372039

[CIT0035] Fechner, D., Reid, S., & Dolnicar, S. (2023). Tourism and emerging infectious diseases: More connections than first meet the eye. *Journal of Travel Research*, *62*(5), 935–948. 10.1177/00472875221127718

[CIT0036] Fehér, A., Gazdecki, M., Véha, M., Szakály, M., & Szakály, Z. (2020). A comprehensive review of the benefits of and the barriers to the switch to a plant-based diet. *Sustainability*, *12*(10), 4136. 10.3390/su12104136

[CIT0037] Garnett, E. E., Balmford, A., Marteau, T. M., Pilling, M. A., & Sandbrook, C. (2021). Price of change: Does a small alteration to the price of meat and vegetarian options affect their sales? *Journal of Environmental Psychology*, *75*, 101589. 10.1016/j.jenvp.2021.101589

[CIT0038] Garnett, E. E., Marteau, T. M., Sandbrook, C., Pilling, M. A., & Balmford, A. (2020). Order of meals at the counter and distance between options affect student cafeteria vegetarian sales. *Nature Food*, *1*(8), 485–488. 10.1038/s43016-020-0132-8 37128072

[CIT0039] Giacalone, D., Clausen, M. P., & Jaeger, S. R. (2022). Understanding barriers to consumption of plant-based foods and beverages: Insights from sensory and consumer science. *Current Opinion in Food Science*, *48*, 100919. 10.1016/j.cofs.2022.100919

[CIT0040] Gómez-Luciano, C. A., de Aguiar, L. K., Vriesekoop, F., & Urbano, B. (2019). Consumers’ willingness to purchase three alternatives to meat proteins in the United Kingdom, Spain, Brazil and the Dominican Republic. *Food Quality and Preference*, *78*, 103732. 10.1016/j.foodqual.2019.103732

[CIT0041] Good Food Institute. (2023). Reducing the price of alternative proteins. Retrieved December 7, 2023, from https://gfi.org/reducing-the-price-of-alternative-proteins/

[CIT0042] Götze, F., & Brunner, T. A. (2021). A consumer segmentation study for meat and meat alternatives in Switzerland. *Foods (Basel, Switzerland)*, *10*(6), 1273. 10.3390/foods10061273 34204963 PMC8229998

[CIT0043] Govaert, G., & Nadif, M. (2008). Block clustering with Bernoulli mixture models: Comparison of different approaches. *Computational Statistics & Data Analysis*, *52*(6), 3233–3245. 10.1016/j.csda.2007.09.007

[CIT0044] Graça, J., Godinho, C. A., & Truninger, M. (2019). Reducing meat consumption and following plant-based diets: Current evidence and future directions to inform integrated transitions. *Trends in Food Science & Technology*, *91*, 380–390. 10.1016/j.tifs.2019.07.046

[CIT0045] Greene, D., Nguyen, M., & Dolnicar, S. (2023). How to entice restaurant patrons to order low-emissions meals? A meta-analysis and research agenda. *Appetite*, *188*, 106612. 10.1016/j.appet.2023.106612 37286169

[CIT0046] Greene, D., Zhu, O. Y., & Dolnicar, S. (2023). Vegan burger, no thanks! Juicy American burger, yes please! The effect of restaurant meal names on affective appeal. *Food Quality and Preference*, *113*, 105042. 10.1016/j.foodqual.2023.105042

[CIT0047] Guthman, J., Butler, M., Martin, S. J., Mather, C., & Biltekoff, C. (2022). In the name of protein. *Nature Food*, *3*(6), 391–393. 10.1038/s43016-022-00532-9 37118035

[CIT0048] Hartmann, C., & Siegrist, M. (2017). Consumer perception and behaviour regarding sustainable protein consumption: A systematic review. *Trends in Food Science & Technology*, *61*, 11–25. 10.1016/j.tifs.2016.12.006

[CIT0049] He, J., Evans, N. M., Liu, H., & Shao, S. (2020). A review of research on plant-based meat alternatives: Driving forces, history, manufacturing, and consumer attitudes. *Comprehensive Reviews in Food Science and Food Safety*, *19*(5), 2639–2656. 10.1111/1541-4337.12610 33336979

[CIT0050] Hoek, A. C., Luning, P. A., Stafleu, A., & de Graaf, C. (2004). Food-related lifestyle and health attitudes of Dutch vegetarians, non-vegetarian consumers of meat substitutes, and meat consumers. *Appetite*, *42*(3), 265–272. 10.1016/j.appet.2003.12.003 15183917

[CIT0051] Hoek, A. C., Luning, P. A., Weijzen, P., Engels, W., Kok, F. J., & de Graaf, C. (2011a). Replacement of meat by meat substitutes. A survey on person- and product-related factors in consumer acceptance. *Appetite*, *56*(3), 662–673. 10.1016/j.appet.2011.02.001 21315123

[CIT0052] Hogan, L. (2018). Food demand in Australia: Trends and issues 2018. Retrieved May 15, 2023, from https://www.agriculture.gov.au/abares/research-topics/food-demand/trends-and-issues-2018

[CIT0053] Humpenöder, F., Bodirsky, B. L., Weindl, I., Lotze-Campen, H., Linder, T., & Popp, A. (2022). Projected environmental benefits of replacing beef with microbial protein. *Nature*, *605*(7908), 90–96. 10.1038/s41586-022-04629-w 35508780

[CIT0054] Iovleff, S., & Singh Bhatia, P. (2023). blockcluster: Co-clustering package for binary, categorical, contingency and continuous data-sets. R package version 4.5.1. Retrieved December 7, 2023, from https://CRAN.R-project.org/package=blockcluster

[CIT0055] IPSOS. (2018). An exploration into diets around the world. Retrieved May 15, 2023, from https://www.ipsos.com/sites/default/files/ct/news/documents/2018-09/an_exploration_into_diets_around_the_world.pdf

[CIT0056] Jahn, S., Furchheim, P., & Strässner, A.-M. (2021). Plant-based meat alternatives: Motivational adoption barriers and solutions. *Sustainability*, *13*(23), 13271. 10.3390/su132313271

[CIT0057] Jęczmyk, A., Uglis, J., & Steppa, R. (2021). Can animals be the key to the development of tourism: A case study of livestock in agritourism. *Animals*, *11*(8), 2357. 10.3390/ani11082357 34438814 PMC8388776

[CIT0058] Joseph, P., Searing, A., Watson, C., & McKeague, J. (2020). Alternative proteins: Market research on consumer trends and emerging landscape. *Meat and Muscle Biology*, *4*(2), 1–11. 10.22175/mmb.11225

[CIT0059] Kahneman, D., Diener, E., & Schwarz, N. (1999). *Well-being: Foundations of hedonic psychology*. Russell Sage Foundation.

[CIT0060] Kaljonen, M., Kortetmäki, T., Tribaldos, T., Huttunen, S., Karttunen, K., Maluf, R. S., Niemi, J., Saarinen, M., Salminen, J., Vaalavuo, M., & Valsta, L. (2021). Justice in transitions: Widening considerations of justice in dietary transition. *Environmental Innovation and Societal Transitions*, *40*, 474–485. 10.1016/j.eist.2021.10.007

[CIT0061] Kemper, J. A., & White, S. K. (2021). Young adults’ experiences with flexitarianism: The 4Cs. *Appetite*, *160*, 105073. 10.1016/j.appet.2020.105073 33359236

[CIT0062] Kerslake, E., Kemper, J. A., & Conroy, D. (2022). What’s your beef with meat substitutes? Exploring barriers and facilitators for meat substitutes in omnivores, vegetarians, and vegans. *Appetite*, *170*, 105864. 10.1016/j.appet.2021.105864 34920050

[CIT0063] Knaapila, A., Michel, F., Jouppila, K., Sontag-Strohm, T., & Piironen, V. (2022). Millennials’ consumption of and attitudes toward meat and plant-based meat alternatives by consumer segment in Finland. *Foods (Basel, Switzerland)*, *11*(3), 456. 10.3390/foods11030456 35159606 PMC8834568

[CIT0064] Kozicka, M., Havlík, P., Valin, H., Wollenberg, E., Deppermann, A., Leclère, D., Lauri, P., Moses, R., Boere, E., Frank, S., Davis, C., Park, E., & Gurwick, N. (2023). Feeding climate and biodiversity goals with novel plant-based meat and milk alternatives. *Nature Communications*, *14*(1), 5316. 10.1038/s41467-023-40899-2 PMC10497520 37699877

[CIT0065] Kuhmonen, I., & Siltaoja, M. (2022). Farming on the margins: Just transition and the resilience of peripheral farms. *Environmental Innovation and Societal Transitions*, *43*, 343–357. 10.1016/j.eist.2022.04.011

[CIT0066] Kumar, P., Chatli, M. K., Mehta, N., Singh, P., Malav, O. P., & Verma, A. K. (2017). Meat analogues: Health promising sustainable meat substitutes. *Critical Reviews in Food Science and Nutrition*, *57*(5), 923–932. 10.1080/10408398.2014.939739 25898027

[CIT0067] Lehtonen, H., Huan-Niemi, E., & Niemi, J. (2022). The transition of agriculture to low carbon pathways with regional distributive impacts. *Environmental Innovation and Societal Transitions*, *44*, 1–13. 10.1016/j.eist.2022.05.002

[CIT0068] Lemken, D., & Langen, N. (2023). The price penalty on meat substitutes—Consumers prefer reduced meat portions over novel meat alternatives and authentic vegetarian dishes—Final stage of a registered report. *Q Open*, *3*(1), 1–27. 10.1093/qopen/qoad009

[CIT0069] Lemken, D., Spiller, A., & Schulze-Ehlers, B. (2019). More room for legume–Consumer acceptance of meat substitution with classic, processed and meat-resembling legume products. *Appetite*, *143*, 104412. 10.1016/j.appet.2019.104412 31445994

[CIT0070] Liu, P., & Tse, E. C.-Y. (2018). Exploring factors on customers’ restaurant choice: An analysis of restaurant attributes. *British Food Journal*, *120*(10), 2289–2303. 10.1108/BFJ-10-2017-0561

[CIT0071] Malan, H., Bartolotto, C., Wilcots, C., Angelis, P., Ferrone, A., Wible, C., Westbrook, E., Fabris, E., Wang, M. C., Slusser, W., Jay, J. A., & Prelip, M. L. (2022). Increasing the selection of low-carbon-footprint entrées through the addition of new menu items and a social marketing campaign in university dining. *Journal of the Association for Consumer Research*, *7*(4), 461–470. 10.1086/720450

[CIT0072] Martin-Rios, C., Rogenhofer, J., & Alvarado, M. S. (2023). The true cost of food waste: Tackling the managerial challenges of the food supply chain. *Trends in Food Science & Technology*, *131*, 190–195. 10.1016/j.tifs.2022.12.005

[CIT0073] Martins, Y., & Pliner, P. (2005). Human food choices: An examination of the factors underlying acceptance/rejection of novel and familiar animal and nonanimal foods. *Appetite*, *45*(3), 214–224. 10.1016/j.appet.2005.08.002 16188344

[CIT0074] Mason-D’Croz, D., Barnhill, A., Bernstein, J., Bogard, J., Dennis, G., Dixon, P., Fanzo, J., Herrero, M., McLaren, R., Palmer, J., Rieder, T., Rimmer, M., & Faden, R. (2022). Ethical and economic implications of the adoption of novel plant-based beef substitutes in the USA: A general equilibrium modelling study. *The Lancet. Planetary Health*, *6*(8), e658–e669. 10.1016/S2542-5196(22)00169-3 35932786 PMC9364141

[CIT0075] Mazac, R., Meinilä, J., Korkalo, L., Järviö, N., Jalava, M., & Tuomisto, H. L. (2022). Incorporation of novel foods in European diets can reduce global warming potential, water use and land use by over 80%. *Nature Food*, *3*(4), 286–293. 10.1038/s43016-022-00489-9 37118200

[CIT0076] McDonagh, L. K., Saunders, J. M., Cassell, J., Curtis, T., Bastaki, H., Hartney, T., & Rait, G. (2018). Application of the COM-B model to barriers and facilitators to chlamydia testing in general practice for young people and primary care practitioners: A systematic review. *Implementation Science*, *13*(1), 1–19. 10.1186/s13012-018-0821-y 30348165 PMC6196559

[CIT0077] Mertens, A., & Oberhoff, L. (2023). Meat-eating justification when gender identity is threatened–The association between meat and male masculinity. *Food Quality and Preference*, *104*, 104731. 10.1016/j.foodqual.2022.104731

[CIT0078] Michalke, A., Köhler, S., Messmann, L., Thorenz, A., Tuma, A., & Gaugler, T. (2023). True cost accounting of organic and conventional food production. *Journal of Cleaner Production*, *408*, 137134. 10.1016/j.jclepro.2023.137134

[CIT0079] Michel, F., Hartmann, C., & Siegrist, M. (2021). Consumers’ associations, perceptions and acceptance of meat and plant-based meat alternatives. *Food Quality and Preference*, *87*, 104063. 10.1016/j.foodqual.2020.104063

[CIT0080] Michie, S., Van Stralen, M. M., & West, R. (2011). The behaviour change wheel: A new method for characterising and designing behaviour change interventions. *Implementation Science*, *6*(1), 1–12. 10.1186/1748-5908-6-42 21513547 PMC3096582

[CIT0081] Mouat, M. J., Prince, R., & Roche, M. M. (2019). Making value out of ethics: The emerging economic geography of lab-grown meat and other animal-free food products. *Economic Geography*, *95*(2), 136–158. 10.1080/00130095.2018.1508994

[CIT0082] Murphy, S. P., Cannon, S. M., & Walsh, L. (2022). Just transition frames: Recognition, representation, and distribution in Irish beef farming. *Journal of Rural Studies*, *94*, 150–160. 10.1016/j.jrurstud.2022.06.009

[CIT0083] Nabuurs, G.-J., Mrabet, R., Abu Hatab, A., Bustamante, M., Clark, H., Havlík, P., House, J., Mbow, C., Ninan, K. N., Popp, A., Roe, S., Sohngen, B., & Towprayoon, S. (2022). Agriculture, forestry and other land uses. In P. R. Shukla, J. Skea, R. Slade, A. Al Khourdajie, R. van Diemen, D. McCollum, M. Pathak, S. Some, P. Vyas, R. Fradera, M. Belkacemi, A. Hasija, G. Lisboa, S. Luz, & J. Malley (Eds.), *IPCC, 2022: Climate change 2022: Mitigation of climate change. Contribution of working group III to the sixth assessment report of the Intergovernmental Panel on Climate Change*. Cambridge University Press.

[CIT0084] Nguyen, J., Ferraro, C., Sands, S., & Luxton, S. (2022). Alternative protein consumption: A systematic review and future research directions. *International Journal of Consumer Studies*, *46*(5), 1691–1717. 10.1111/ijcs.12797

[CIT0085] Niva, M., & Vainio, A. (2021). Towards more environmentally sustainable diets? Changes in the consumption of beef and plant-and insect-based protein products in consumer groups in Finland. *Meat Science*, *182*, 108635. 10.1016/j.meatsci.2021.108635 34303133

[CIT0086] Nutrition Australia. (2021). Australian Dietary Guidelines: Recommended daily intakes. Retrieved May 15, 2023, from https://nutritionaustralia.org/fact-sheets/adgs-recommended-daily-intakes/

[CIT0087] Onwezen, M. C., Bouwman, E. P., Reinders, M. J., & Dagevos, H. (2021). A systematic review on consumer acceptance of alternative proteins: Pulses, algae, insects, plant-based meat alternatives, and cultured meat. *Appetite*, *159*, 105058. 10.1016/j.appet.2020.105058 33276014

[CIT0088] Ortega, D. L., Sun, J., & Lin, W. (2022). Identity labels as an instrument to reduce meat demand and encourage consumption of plant based and cultured meat alternatives in China. *Food Policy*, *111*, 102307. 10.1016/j.foodpol.2022.102307

[CIT0089] Ozdemir, B., & Caliskan, O. (2015). Menu design: A review of literature. *Journal of Foodservice Business Research*, *18*(3), 189–206. 10.1080/15378020.2015.1051428

[CIT0090] Pohjolainen, P., Vinnari, M., & Jokinen, P. (2015). Consumers’ perceived barriers to following a plant-based diet. *British Food Journal*, *117*(3), 1150–1167. 10.1108/BFJ-09-2013-0252

[CIT0091] Ponnam, A., & Balaji, M. (2014). Matching visitation-motives and restaurant attributes in casual dining restaurants. *International Journal of Hospitality Management*, *37*, 47–57. 10.1016/j.ijhm.2013.10.004

[CIT0092] Poore, J., & Nemecek, T. (2018). Reducing food’s environmental impacts through producers and consumers. *Science (New York, NY)*, *360*(6392), 987–992. 10.1126/science.aaq0216 29853680

[CIT0093] Ritchie, H., & Roser, M. (2019). Meat and dairy production. Retrieved May 15, 2023, from https://ourworldindata.org/meat-production

[CIT0094] Rubio, N. R., Xiang, N., & Kaplan, D. L. (2020). Plant-based and cell-based approaches to meat production. *Nature Communications*, *11*(1), 6276. 10.1038/s41467-020-20061-y PMC7722853 33293564

[CIT0095] Saget, S., Costa, M., Santos, C. S., Vasconcelos, M. W., Gibbons, J., Styles, D., & Williams, M. (2021). Substitution of beef with pea protein reduces the environmental footprint of meat balls whilst supporting health and climate stabilisation goals. *Journal of Cleaner Production*, *297*, 126447. 10.1016/j.jclepro.2021.126447

[CIT0096] Saksena, M. J., Okrent, A. M., Anekwe, T. D., Cho, C., Dicken, C., Effland, A., Elitzak, H., Guthrie, J., Hamrick, K. S., & Hyman, J. (2018). America’s eating habits: Food away from home. Retrieved May 15, 2023, from https://ideas.repec.org/p/ags/uersib/281119.html

[CIT0097] Santo, R. E., Kim, B. F., Goldman, S. E., Dutkiewicz, J., Biehl, E., Bloem, M. W., Neff, R. A., & Nachman, K. E. (2020). Considering plant-based meat substitutes and cell-based meats: A public health and food systems perspective. *Frontiers in Sustainable Food Systems*, *4*, 134. 10.3389/fsufs.2020.00134

[CIT0098] Seo, S., & Lee, H. (2017). What makes restaurateurs adopt healthy restaurant initiatives? *British Food Journal*, *119*(12), 2583–2596. 10.1108/BFJ-06-2016-0285

[CIT0099] Sexton, A. E., Garnett, T., & Lorimer, J. (2019). Framing the future of food: The contested promises of alternative proteins. *Environment and Planning. E, Nature and Space*, *2*(1), 47–72. 10.1177/2514848619827009 PMC6989034 32039343

[CIT0101] Souza Filho, P. F., Andersson, D., Ferreira, J. A., & Taherzadeh, M. J. (2019). Mycoprotein: Environmental impact and health aspects. *World Journal of Microbiology & Biotechnology*, *35*(10), 147. 10.1007/s11274-019-2723-9 31549247 PMC6757021

[CIT0102] Sparkman, G., Weitz, E., Robinson, T. N., Malhotra, N., & Walton, G. M. (2020). Developing a scalable dynamic norm menu-based intervention to reduce meat consumption. *Sustainability*, *12*(6), 2453. 10.3390/su12062453

[CIT0103] Stoll-Kleemann, S., & Schmidt, U. J. (2017). Reducing meat consumption in developed and transition countries to counter climate change and biodiversity loss: A review of influence factors. *Regional Environmental Change*, *17*(5), 1261–1277. 10.1007/s10113-016-1057-5

[CIT0104] Szenderák, J., Fróna, D., & Rákos, M. (2022). Consumer acceptance of plant-based meat substitutes: A narrative review. *Foods (Basel, Switzerland)*, *11*(9), 1274. 10.3390/foods11091274 35563997 PMC9102955

[CIT0105] Taufik, D., Bouwman, E. P., Reinders, M. J., & Dagevos, H. (2022). A reversal of defaults: Implementing a menu-based default nudge to promote out-of-home consumer adoption of plant-based meat alternatives. *Appetite*, *175*, 106049. 10.1016/j.appet.2022.106049 35460809

[CIT0106] Tay, W., Quek, R., Lim, J., Kaur, B., Ponnalagu, S., & Henry, C. J. (2023). Plant-based alternative proteins—are they nutritionally more advantageous? *European Journal of Clinical Nutrition*, *77*(11), 1051–1060. 10.1038/s41430-023-01328-1 37580584

[CIT0107] The R Foundation. (2023). *The R project for statistical computing*. Retrieved December 7, 2023, from https://www.r-project.org/

[CIT0108] Toh, D. W. K., Srv, A., & Henry, C. J. (2022). Unknown impacts of plant-based meat alternatives on long-term health. *Nature Food*, *3*(2), 90–91. 10.1038/s43016-022-00463-5 37117959

[CIT0109] Turnwald, B. P., Anderson, K. G., Jurafsky, D., & Crum, A. J. (2020). Five-star prices, appealing healthy item descriptions? Expensive restaurants’ descriptive menu language. *Health Psychology: Official Journal of the Division of Health Psychology, American Psychological Association*, *39*(11), 975–985. 10.1037/hea0001025 32940527

[CIT0110] United Nations Environment Programme. (2021). Closing the gap – executive brief. Retrieved April 17, 2023, from https://wedocs.unep.org/handle/20.500.11822/35853

[CIT0111] Vallikkadan, M. S., Dhanapal, L., Dutta, S., Sivakamasundari, S., Moses, J., & Anandharamakrishnan, C. (2023). Meat alternatives: Evolution, structuring techniques, trends, and challenges. *Food Engineering Reviews*, *15*(2), 329–359. 10.1007/s12393-023-09332-8

[CIT0112] Van Loo, E. J., Caputo, V., & Lusk, J. L. (2020). Consumer preferences for farm-raised meat, lab-grown meat, and plant-based meat alternatives: Does information or brand matter? *Food Policy*. *95*, 101931. 10.1016/j.foodpol.2020.101931

[CIT0113] Vandenbroele, J., Slabbinck, H., Van Kerckhove, A., & Vermeir, I. (2021). Mock meat in the butchery: Nudging consumers toward meat substitutes. *Organizational Behavior and Human Decision Processes*, *163*, 105–116. 10.1016/j.obhdp.2019.09.004

[CIT0114] Verhoeven, A. A., Adriaanse, M. A., de Vet, E., Fennis, B. M., & de Ridder, D. T. (2015). It’s my party and I eat if I want to. Reasons for unhealthy snacking. *Appetite*, *84*, 20–27. 10.1016/j.appet.2014.09.013 25261101

[CIT0115] Weinrich, R. (2019). Opportunities for the adoption of health-based sustainable dietary patterns: A review on consumer research of meat substitutes. *Sustainability*, *11*(15), 4028. 10.3390/su11154028

[CIT0116] Wood, W., & Rünger, D. (2016). Psychology of habit. *Annual Review of Psychology*, *67*(1), 289–314. 10.1146/annurev-psych-122414-033417 26361052

[CIT0117] World Resources Institute. (2020). Playbook for guiding diners toward plant-rich dishes in food service. Retrieved May 15, 2023, from https://www.wri.org/research/playbook-guiding-diners-toward-plant-rich-dishes-food-service

[CIT44811432] Xu, Y., & Jeong, E. (2019). The effect of message framings and green practices on customers’ attitudes and behavior intentions toward green restaurants. *International Journal of Contemporary Hospitality Management*, *31*(6), 2270–2296. doi:10.1108/IJCHM-05-2018-0386

[CIT0118] Ye, T., & Mattila, A. S. (2021). The effect of ad appeals and message framing on consumer responses to plant-based menu items. *International Journal of Hospitality Management*, *95*, 102917. 10.1016/j.ijhm.2021.102917

[CIT0119] Ye, T., & Mattila, A. S. (2022). The impact of environmental messages on consumer responses to plant-based meat: Does language style matter? *International Journal of Hospitality Management*, *107*, 103298. 10.1016/j.ijhm.2022.103298

