# Supplementary material for: Identifying segment-specific barriers to ordering environmentally sustainable plant-based meat dishes in restaurants
Source: J Sustain Tour. 2024 Apr 22;33(2):333–56. doi: 10.1080/09669582.2024.2342982 (PMC11774254; doi:10.1080/09669582.2024.2342982)
Supplement: Supplemental Material [file RSUS_A_2342982_SM6180.docx]

**Appendix A**

**Overview of existing plant-based meat segmentation studies**

| **Author** | **Topic / Country (sample size) / Data analysis** | **Segmentation variables** | **Descriptor variables** | **Results** |
| --- | --- | --- | --- | --- |
| Apostolidis and McLeay (2016) | Plant-based meat /  UK (n=247) /  Latent class analysis | Importance of fat content, carbon footprint, type of mince, method of production, price, origin of plant-based meats | Gender, age, income, household with children, region of residence, meat consumption frequency | Cluster 1: Price conscious (42.5%)  Cluster 2: Healthy eaters (10.5%)  Cluster 3: Taste driven (14.6%)  Cluster 4: Green (17%)  Cluster 5: Organic (9.7%)  Cluster 6: Vegetarian (5.7%) |
| Beacom et al. (2021) | Dairy and meat alternatives /  UK and Ireland (n=456) /  Logistic regression | Consumption of dairy and meat alternatives | Age, gender, education, income, region of residence | Cluster 1: Consumers of dairy and meat alternatives (88%)  Cluster 2: Non-consumers of dairy and meat alternatives (12%)  Females and those living in urban areas were more likely to consume dairy and meat alternatives. |
| Cardello et al. (2022) | Plant-based milks /  New Zealand (n=345) /  Penalty/lift analysis | Product liking | Frequency of consumption, purchase frequency, sensory attributes, emotional, cognitive, and situational terms | Cluster 1: Full-fat dairy likers (31%)  Cluster 2: Dairy and tri-blend likers (16%)  Cluster 3: Full-fat dairy / tri-blend / soy Likers (21%)  Cluster 4: Plant-based likers (32%) |
| Escribano et al. (2021) | Plant-based and cultured meat / Spain (n= 444) /  k-means cluster analysis | Age, gender, education, income, household size, meat consumption frequency, place of purchase of meat, responsibility for household food purchases, purchase of plant-based food frequency, lifestyle, perception of meat, meat attributes |  | Cluster 1: Price-sensitive millennials  Cluster 2: Conscious/concerned consumers  Cluster 3: Indifferent consumers |
| Götze and Brunner (2021) | Meat alternatives /  Switzerland (n=561) /  Hierarchical cluster analysis | Habit of eating meat, attitude towards meat alternatives, preferences for replacing meat, meat importance for health, meat unimportance for health, environmental impact of meat, meat reduction intention, no renunciation of meat | Gender, age, area of residence, household size, number of children, education, employment, income, nutrition knowledge, body mass index, diet- and food consumption-related attitudes, meat alternative consumption, motives and barriers to eat meat alternatives, | Cluster 1: Environmentally and health-oriented meat-eaters (16.8%) Cluster 2: Uncompromising meat-eaters (18.1%),  Cluster 3: Moderate meat-eaters who are willing to replace meat (15.9%) Cluster 4: Indifferent but moderate meat-eaters (21.2%) Cluster 5: Environmentally conscious regular meat-eaters (13.7%)  Cluster 6: Environmentally and health-conscious meat avoiders (14.4%) |
| Gómez-Luciano et al. (2019) | Meat alternatives /  UK (n=180), Brazil (n=216), Spain (n=200), Dominican Republic (n=133) /  Logistic regression | Willingness to buy plant-based meat | Food neophobia, food technology neophobia, healthiness, safety, price of plant-based meat, environmental impact of foods, convenience | UK/Spain/Brazil: Healthiness, safety, and nutritional characteristics are most important.  Dominican Republic: food technology neophobia is most important. |
| Hoek et al. (2004) | Meat substitutes /  Netherland (n=4415) /  Logistic regression | Dietary lifestyle (vegetarians, meat substitute eaters, meat eaters) | Gender, age, education, income, area of residence, household size, food-related lifestyle, health consciousness | Vegetarians (n=63) and consumers of meat substitutes (n=39): higher education levels, higher social economic status, smaller households, and more urbanised residential areas, compared to meat consumers (n=4313). |
| Hoek et al. (2011a) | Meat substitutes /  UK (n=235), Netherlands (n=318) /  t-tests, ANOVA and chi-square-tests, Kendall’s tau-b correlation coefficient | Consumption frequency of meat alternatives | Age, gender, household size including children, education, food neophobia, food choice motives, attitudes and beliefs towards meat and meat substitutes, desired new meat substitute attributes | Non-users (n = 324) and light/medium-users (n = 133): Barriers include unfamiliarity with meat substitutes and the lower sensory attractiveness.  Only heavy-users (n=96) were motivated to choose ethical foods. |
| Knaapila et al. (2022) | Plant-based meat /  Finland (n=550) /  independent samples t-test, ANOVA, Chi-square test | Associations with meat and meat alternatives | Dietary lifestyle, education, age, gender, meat consumption frequency, meat alternative consumption frequency, reasons for eating meat plant-based meat, barriers for eating plant-based meat alternatives, importance of meat in main meals, diet-related attitudes (health consciousness, ecological welfare, natural content, food neophobia, meat commitment) | Cluster 1: Meat positive (14.3%)  Cluster 2: Meat preference (20%)  Cluster 3: Both positive (23.6%)  Cluster 4: No positive (10.6%)  Cluster 5: Meat alternative preference (13.9%)  Cluster 6: Meat alternative positive (17.6%) |
| Lemken et al. (2019) | Plant-based meat /  Germany (GER, n=633), New Zealand (NZ, n=445) /  Latent class analysis | Acceptance to replace meat with legumes, use processed legumes and use meat substitutes | Meat consumption frequency, interest in legumes, healthiness of meat, vegetarian friends, dramatic relief on germs, food neophobia, gender, age, income, size of origin region, size of living area, number of children, connection to agriculture | Cluster 1: Only meat (24.5% in NZ, 30.0% in GER) Cluster 2: Meat first (14.8% in NZ, 16.8% in GER) Cluster 3: The pioneers (18.2% in NZ, 14.4% in GER) Cluster 4: Open but wary to replace (17.1% in NZ) /Wary of processed (16.8% in GER)  Cluster 5: Seekers of meat alternatives (25.4% in NZ) / Contemplators (22.1% in GER) |
| Niva and Vainio (2021) | Plant-based meat /  Finland (n=1000) /  Latent class analysis | Changes in food consumption of meat and plant-based foods, expected changes in food consumption | Eating motives (price, health, pleasure), food neophobia, sustainability, age, gender, education, size of place of residence, consumption frequencies of different animal and plant-based proteins | Cluster 1: Established beef lovers (37.0%) Cluster 2: Alternative protein increasers (25.5%) Cluster 3: Established ‘light’ flexitarians (20.3%) Cluster 4: Beef-avoiding plant protein increasers (9.3%) Cluster 5: Beef reducers (8.0%) |
| Reipurth et al. (2019) | Plant-based foods /  Denmark (n=462) /  k-means segmentation, logistic regression | Consumption frequency of meat, dairy, fish, eggs | Rating of plant-based foods in cost, convenience, skill of cooking, health, social situations, social support, gender, age, education, area of residence, household size, income | Cluster 1: Low All (n=220, 48%)  Cluster 2: High All (n=70, 15%)  Cluster 3: High Meat (n=48, 10%)  Cluster 4: High Dairy (n=124, 27%) |
| Szejda et al. (2021) | Plant-based and cultivated meat /  South Africa (n=959) / Regression analysis | Purchase intentions | Familiarity, purchasing reasons (animal welfare, health, environment. No pathogens, no antibiotics, local food security, wild animal conservation), age | 17.3% of participants were very or extremely familiar 67% were highly likely to try  59% were highly likely to purchase  60% of born-frees, 62% of millennials, and 53% of Gen X were highly likely to purchase plant-based meat; Animal welfare, health, the environment, and food security were main purchasing reasons. |
| Vainio et al. (2016) | Plant-based foods /  Finland (n=1048) /  Latent class analysis | Past changes and future intentions of consuming beef, beans, and soy products | Gender, age, education, place of residence, diet, consumption of poultry and fish | Beef only (25.4%) Beef and beans (23.2%) Beef, beans, and soy products (23.0%) No beef (7.5%) Ongoing dietary change towards plant proteins (12.1%) Past dietary attempt towards plant proteins (8.1%) |

**Appendix B**

**Questionnaire**

We will now ask you a few questions about plant-based meats.

Plant-based meats are protein-containing foods made from vegetables, mushrooms, and grains – such as soy, peas, or wheat. Plant-based meats aim to be similar in taste, texture, and smell to animal meat and can replace the function of animal meat in meals. However, plant-based meats are different to cultivated meats which are made from animal cells in laboratories.

You may have noticed plant-based meats on restaurant menus. For example, burger joints offer plant-based meat burgers and Asian restaurants use plant-based meats to replace animal meat in traditional dishes like cashew chicken, sweet sour pork, and curries.


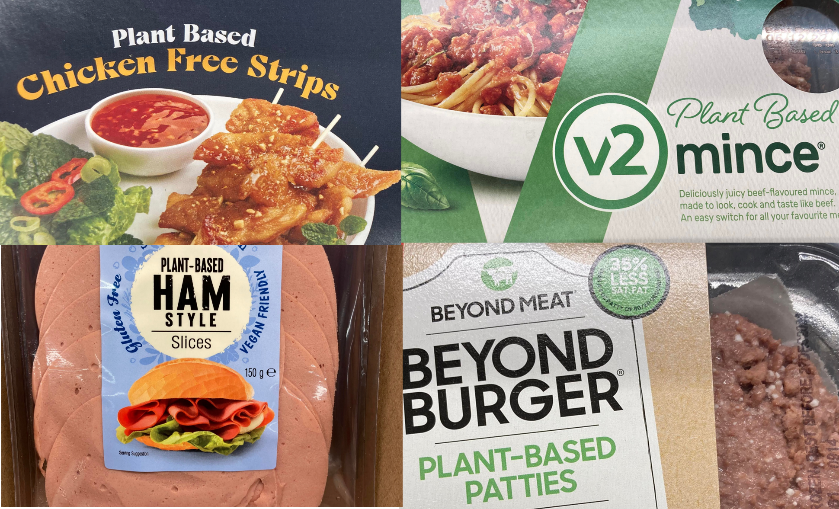


**Have you heard of plant-based meats before?**

Yes

No

**What do you think of restaurants that offer plant-based meat dishes?**

____________________________________________________

**Does your favourite restaurant serve plant-based meat dishes?**

Yes

No

**How often do you order the following dishes when having dinner at a restaurant?**

Animal meat dish

Plant-based meat dish

Vegetarian/vegan dish without plant-based meats

Allocate 100%

Please now imagine having dinner at a casual dining restaurant. You are sitting at the table and are reading the menu. You notice that the restaurant offers plant-based meat dishes.

Over the next four pages, you will see a total of 32 reasons people give for not ordering plant-based meat dishes. Please tell us which of those prevent YOU from ordering a plant-based meat dish


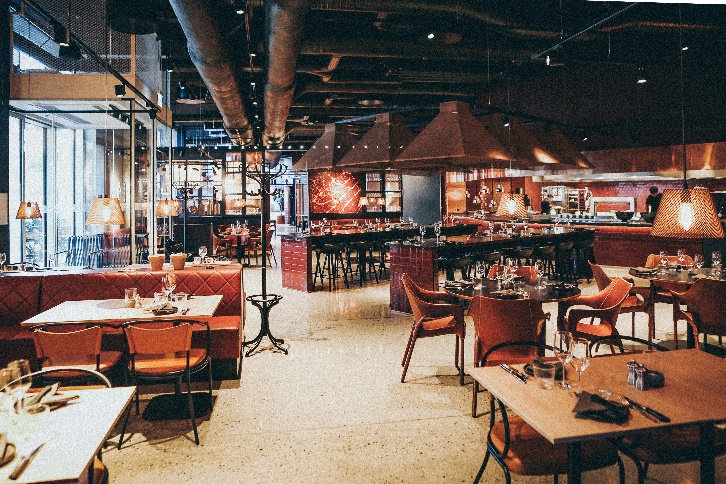


I do not order plant-based meat dishes because… **Agree Disagree (randomised)**

they are expensive.

I love the taste of animal meat.

I do not like the taste of plant-based meats.

I prefer vegan/vegetarian dishes without plant-based meats.

they get boring.

I think they do not contain enough protein.

I think they do not contain enough iron.

I think they do not contain enough fibre.

I think they contain too much soy.

I think they contain too much saturated fat.

I think they contain too much sugar.

I think they contain too much sodium.

I think a complete meal is a meal with animal meat.

I am allergic to plant-based meats.

I think they contain too many genetically modified ingredients.

I need animal meat to feel physically strong.

I do not want to be stigmatised as a vegan/vegetarian.

they are for vegans/vegetarians only.

I think of myself as an animal meat eater.

humans are natural animal meat-eaters.

most of my family and friends order an animal meat dish.

my family and friends expect me to order an animal meat dish.

I don’t trust the technology used to create plant-based meats.

I don’t know what plant-based meats are made of.

I don’t like the idea of mimicking animal meat using plants.

I don’t like that restaurants describe plant-based meat dishes with animal meat-like names.

I worry I will not enjoy them.

someone else orders for me.

I share meals with others who do not want to eat plant-based meats.

animal meat is more environmentally friendly than plant-based meat.

eating an animal meat dish is part of the restaurant experience.

I always eat an animal meat dish.

**When having dinner at a restaurant, how important is it to you to… (randomised)**

eat a delicious meal.

eat a healthy meal.

eat an environmentally sustainable meal.

eat a meal that’s good value for money.

eat foods you usually eat.

not important at all (0) |-------------| extremely important (100)

**How many times a month do you eat at a restaurant?** Only enter numbers and no ranges.

____ times.

**When you have dinner at a restaurant, how much do you pay per person, on average?** Only enter numbers and no ranges.

AUD$....

**When going out for dinner, how often do you go to …**

 Fast food restaurants (counter service, fast delivery, low priced)

 Casual dining restaurants (counter or table service, casual environment, moderately priced)

 Fine dining restaurants (table service, elegant environment, high priced)

Allocate 100%

**How many restaurants do you think exist in Australia? Please select the third answer option to demonstrate that you are reading the questions carefully.**

50,000

55,000

60,000

65,000

**How often do you go out for dinner to…** (The answers DO NOT need to add up to 100% because they are not mutually exclusive) **(randomised)**

spend time with a partner

celebrate a special occasion

socialise with family and friends

eat a meal without needing to cook

0% of the time |-------| 100% of the time

**How often do you go out for dinner …** (The answers DO NOT need to add up to 100% because they are not mutually exclusive) **(randomised)**

alone

with your partner

with your partner and children

with relatives

with friends

with work colleagues

With someone who does not eat animal meat

0% of the time |-------| 100% of the time

**What are your personal hobbies? Yes No**

Board games and card games

Cars and vehicles

Cooking and baking

DIY arts and crafts

Doing sports and fitness

Gardening and plants

Making music

Meditation and wellness

Outdoor activities

Pets

Photography

Reading

Socialising

Tech and computers

Traveling

Video gaming

Writing

**Would you describe yourself as a person who … (randomised)**

cares about the environment.

cares about their fitness.

cares about other people’s opinions.

cares about their health.

loves animals.

knows a lot about plant-based meats.

knows a lot about nutrition.

knows a lot about climate change.

is open to new experiences.

Absolutely not (0) |-------------| Absolutely (100)

**What news stories do you pay attention to? Yes No**

Business, Finance & Economics

Computer, Science & Technology

Current affairs

Education

Entertainment, Art & Culture

Environment

Health & Medicine

Lifestyle & Fashion

Sports

**Where do you get your news from? Yes No**

News websites

Podcasts

Printed newspapers

Radio

Facebook

TikTok

LinkedIn

Twitter

Instagram

Snapchat

TV

**How important do you think the following things are to slow down climate change? (randomised)**

Fly less.

Drive less.

Eat less meat and dairy products.

Reduce waste.

Reduce energy use.

not important at all (0) |-------------| extremely important (100)

**What is your gender?**

Female

Male

Non-binary

Other

**In what year were you born?**

….

**What is your nationality? Dropdown menu with all nationalities.**

**What is your highest level of completed education?**

Primary School

High School

Certificate (e.g., TAFE)

Bachelor’s Degree

Graduate Certificate / Diploma

Postgraduate Degree

No formal education completed

**How many people live in your household?**

….

**What was your household income before taxes in the financial year 2021/2022?**

Prefer not to say

I do not know

<$33,799

$33,800-$41,599

$41,600-$51,999

$52,000-$64,999

$65,000-$77,999

$78,000-$90,999

$91,000-$103,999

$104,000-$155,999

>$156,000 or more

**What is your postcode?**

…..

**Which political party did you vote for in the 2022 Australian federal election?**

Greens

Independent and others

Labor

Liberal-National Coalition

One Nation

United Australia

I did not vote

Prefer not to say

**Do you currently follow any of the following diets? Tick all that apply.**

Carnivore

Keto

Paleo

Vegan

Vegetarian

I do not follow any of these diets

**Appendix C**

**Overview of descriptor variable**

| **Characteristic** | **Total** | **Segment 1** | **Segment 2** | **Segment 3** | **Segment 4** | **Segment 5** | **Segment 6** |
| --- | --- | --- | --- | --- | --- | --- | --- |
| *Age** | 34.6 (0.5) | 31.9 (0.8) | 37.0 (1.3) | 32.5 (0.8) | 39.6 (1.4) | 36.3 (1.6) | 36.8 (2.5) |
| *Household size* | 2.8 (0.1) | 2.8 (0.1) | 2.6 (0.1) | 2.9 (0.1) | 2.8 (0.1) | 2.9 (0.2) | 3.0 (0.3) |
| *Gender** |  |  |  |  |  |  |  |
| Female | 49.9% (1.0%) | 60.0% (1.9%) | 58.2% (2.7%) | 51.5% (1.8%) | 48.4% (2.6%) | 28.7% (2.2%) | 21.7% (3.5%) |
| Male | 48.2% (1.0%) | 38.2% (1.8%) | 39.2% (2.7%) | 45.5% (1.8%) | 51.6% (2.6%) | 71.3% (2.2%) | 73.9% (4.0%) |
| Non-binary | 1.9% (0.1%) | 1.8% (0.1%) | 2.5% (0.3%) | 3.0% (0.2%) | 0.0% (0.0%) | 0.0% (0.0%) | 4.3% (0.9%) |
| *Education** |  |  |  |  |  |  |  |
| Primary School | 0.6% (0.0%) | 0.6% (0.0%) | 0.0% (0.0%) | 1.0% (0.1%) | 1.1% (0.1%) | 0.0% (0.0%) | 0.0% (0.0%) |
| High School | 16.8% (0.6%) | 18.8% (1.2%) | 13.9% (1.3%) | 14.1% (0.9%) | 16.8% (1.4%) | 23.0% (1.9%) | 13.0% (2.4%) |
| Certificate | 13.4% (0.5%) | 17.6% (1.1%) | 6.3% (0.7%) | 12.1% (0.8%) | 9.5% (0.9%) | 16.1% (1.4%) | 26.1% (4.0%) |
| Graduate Certificate / Diploma | 9.6% (0.3%) | 7.9% (0.6%) | 12.7% (1.2%) | 8.1% (0.5%) | 11.6% (1.1%) | 12.6% (1.2%) | 4.3% (0.9%) |
| Bachelor’s Degree | 37.7% (0.9%) | 29.1% (1.6%) | 30.4% (2.4%) | 46.5% (1.8%) | 38.9% (2.4%) | 35.6% (2.5%) | 52.2% (5.2%) |
| Postgraduate Degree | 21.8% (0.7%) | 26.1% (1.5%) | 36.7% (2.6%) | 18.2% (1.1%) | 22.1% (1.8%) | 12.6% (1.2%) | 4.3% (0.9%) |
| *Income* |  |  |  |  |  |  |  |
| $33,800-$41,599 | 4.9% (0.2%) | 4.2% (0.3%) | 5.1% (0.5%) | 5.6% (0.4%) | 2.1% (0.2%) | 4.6% (0.5%) | 17.4% (3.0%) |
| $41,600-$51,999 | 2.6% (0.1%) | 4.8% (0.4%) | 1.3% (0.1%) | 2.5% (0.2%) | 1.1% (0.1%) | 1.1% (0.1%) | 4.3% (0.9%) |
| $52,000-$64,999 | 7.3% (0.3%) | 7.3% (0.5%) | 7.6% (0.8%) | 7.6% (0.5%) | 6.3% (0.6%) | 5.7% (0.6%) | 13.0% (2.4%) |
| $65,000-$77,999 | 7.6% (0.3%) | 8.5% (0.6%) | 8.9% (0.9%) | 6.1% (0.4%) | 10.5% (1.0%) | 6.9% (0.7%) | 0.0% (0.0%) |
| $78,000-$90,999 | 4.9% (0.2%) | 4.2% (0.3%) | 5.1% (0.5%) | 5.6% (0.4%) | 5.3% (0.5%) | 4.6% (0.5%) | 4.3% (0.9%) |
| $91,000-$103,999 | 8.2% (0.3%) | 9.7% (0.7%) | 8.9% (0.9%) | 8.6% (0.6%) | 7.4% (0.7%) | 5.7% (0.6%) | 4.3% (0.9%) |
| $104,000-$155,999 | 17.2% (0.6%) | 16.4% (1.1%) | 17.7% (1.6%) | 17.2% (1.0%) | 12.6% (1.1%) | 23.0% (1.9%) | 17.4% (3.0%) |
| $156,000-$181,999 | 7.3% (0.3%) | 6.7% (0.5%) | 7.6% (0.8%) | 6.1% (0.4%) | 13.7% (1.2%) | 4.6% (0.5%) | 4.3% (0.9%) |
| $182,000-$207,999 | 5.7% (0.2%) | 4.2% (0.3%) | 6.3% (0.7%) | 6.1% (0.4%) | 5.3% (0.5%) | 8.0% (0.8%) | 4.3% (0.9%) |
| $208,000-$233,999 | 2.9% (0.1%) | 1.8% (0.1%) | 8.9% (0.9%) | 1.0% (0.1%) | 1.1% (0.1%) | 4.6% (0.5%) | 8.7% (1.7%) |
| $234,000-$259,999 | 2.8% (0.1%) | 2.4% (0.2%) | 2.5% (0.3%) | 3.0% (0.2%) | 2.1% (0.2%) | 4.6% (0.5%) | 0.0% (0.0%) |
| $260,000-$311,999 | 2.5% (0.1%) | 3.6% (0.3%) | 1.3% (0.1%) | 1.0% (0.1%) | 2.1% (0.2%) | 5.7% (0.6%) | 0.0% (0.0%) |
| $312,000-$415,999 | 0.8% (0.0%) | 2.4% (0.2%) | 0.0% (0.0%) | 0.5% (0.0%) | 0.0% (0.0%) | 0.0% (0.0%) | 0.0% (0.0%) |
| $416,000 or more | 0.5% (0.0%) | 1.2% (0.1%) | 0.0% (0.0%) | 0.5% (0.0%) | 0.0% (0.0%) | 0.0% (0.0%) | 0.0% (0.0%) |
| I do not know | 6.3% (0.2%) | 10.3% (0.7%) | 3.8% (0.4%) | 6.1% (0.4%) | 5.3% (0.5%) | 4.6% (0.5%) | 0.0% (0.0%) |
| Prefer not to say | 10.2% (0.4%) | 4.8% (0.4%) | 10.1% (1.0%) | 12.1% (0.8%) | 15.8% (1.4%) | 9.2% (0.9%) | 13.0% (2.4%) |
| Missing | 8.3% (0.3%) | 7.3% (0.5%) | 5.1% (0.5%) | 10.6% (0.7%) | 9.5% (0.9%) | 6.9% (0.7%) | 8.7% (1.7%) |
| *Number of restaurant visits per month* | 3.6 (0.1) | 3.4 (0.2) | 3.5 (0.4) | 3.7 (0.2) | 3.6 (0.3) | 3.9 (0.3) | 4.2 (1.0) |
| *Average spending per person per restaurant visit* | 37.2 (0.7) | 34.9 (1.1) | 38.5 (2.0) | 37.0 (1.3) | 39.6 (1.8) | 38.4 (2.1) | 35.9 (4.6) |
| *Diet** |  |  |  |  |  |  |  |
| Carnivore | 7.4% (0.3%) | 3.0% (0.2%) | 0.0% (0.0%) | 9.1% (0.6%) | 7.4% (0.7%) | 16.1% (1.4%) | 17.4% (3.0%) |
| Keto | 2.6% (0.1%) | 1.8% (0.1%) | 1.3% (0.1%) | 2.0% (0.1%) | 7.4% (0.7%) | 1.1% (0.1%) | 4.3% (0.9%) |
| None | 77.9% (0.7%) | 64.2% (1.8%) | 81.0% (1.7%) | 85.9% (0.9%) | 78.9% (1.7%) | 82.8% (1.5%) | 73.9% (4.0%) |
| Paleo | 0.8% (0.0%) | 0.6% (0.0%) | 0.0% (0.0%) | 0.5% (0.0%) | 2.1% (0.2%) | 0.0% (0.0%) | 4.3% (0.9%) |
| Vegetarian | 8.8% (0.3%) | 23.6% (1.4%) | 12.7% (1.2%) | 2.0% (0.1%) | 4.2% (0.4%) | 0.0% (0.0%) | 0.0% (0.0%) |
| Vegan | 2.5% (0.1%) | 6.7% (0.5%) | 5.1% (0.5%) | 0.5% (0.0%) | 0.0% (0.0%) | 0.0% (0.0%) | 0.0% (0.0%) |
| *Food ordering* |  |  |  |  |  |  |  |
| Meat* | 68.6 (1.3) | 47.4 (2.9) | 49.5 (3.6) | 76.8 (1.6) | 77.7 (2.4) | 91.6 (1.2) | 90.3 (1.7) |
| Plant-based meats* | 7.3 (0.6) | 15.2 (1.6) | 8.0 (1.6) | 4.8 (0.6) | 5.6 (1.4) | 1.3 (0.4) | 1.2 (0.5) |
| Vegetarian* | 24.1 (1.0) | 37.4 (2.2) | 42.5 (3.2) | 18.4 (1.4) | 16.7 (1.8) | 7.1 (1.0) | 8.5 (1.4) |
| *Type of restaurant* | |  |  |  |  |  |  |
| Fast-food* | 34.9 (1.1) | 34.5 (2.1) | 26.7 (3.1) | 37.2 (2.0) | 33.2 (2.9) | 36.9 (3.0) | 44.8 (4.9) |
| Casual | 53.2 (1.0) | 56.0 (1.9) | 57.0 (3.2) | 51.9 (1.9) | 52.5 (2.7) | 51.0 (2.8) | 43.3 (4.7) |
| Fine dining* | 11.9 (0.6) | 9.5 (1.0) | 16.3 (2.5) | 10.8 (1.0) | 14.3 (1.8) | 12.1 (1.4) | 12.0 (3.1) |
| *Motives to visit a restaurant* | |  |  |  |  |  |  |
| With partner | 52.7 (1.4) | 50.7 (2.8) | 53.8 (4.2) | 49.0 (2.6) | 57.7 (3.4) | 56.9 (3.9) | 57.0 (7.0) |
| Celebrate | 55.8 (1.1) | 54.8 (2.2) | 59.1 (3.1) | 55.7 (2.0) | 56.5 (2.9) | 54.1 (3.0) | 56.7 (6.5) |
| Social | 67.2 (1.0) | 66.4 (2.0) | 67.7 (3.1) | 67.4 (1.8) | 68.3 (2.7) | 67.4 (3.0) | 62.9 (6.1) |
| Eat | 56.5 (1.2) | 53.2 (2.3) | 54.6 (3.4) | 57.0 (2.1) | 54.5 (3.2) | 63.1 (2.7) | 65.1 (7.0) |
| *Social context when visiting a restaurant* | | |  |  |  |  |  |
| Alone | 17.2 (1.0) | 16.6 (1.8) | 18.1 (2.8) | 16.5 (1.9) | 17.9 (2.6) | 14.7 (2.5) | 30.5 (6.7) |
| Partner | 54.4 (1.5) | 52.2 (2.9) | 56.4 (4.2) | 50.2 (2.8) | 60.4 (3.7) | 60.3 (4.1) | 53.5 (6.7) |
| Partner and children* | 24.3 (1.4) | 16.3 (2.4) | 21.9 (3.9) | 24.8 (2.6) | 37.2 (4.0) | 23.3 (3.8) | 36.7 (7.8) |
| Relatives | 41.6 (1.2) | 41.6 (2.3) | 39.8 (3.2) | 45.8 (2.1) | 38.4 (2.8) | 40.8 (3.2) | 27.2 (6.0) |
| Friend | 50.5 (1.2) | 50.4 (2.2) | 50.3 (3.4) | 51.7 (2.1) | 50.1 (3.1) | 50.4 (3.1) | 43.7 (6.2) |
| Colleagues | 19.4 (1.0) | 16.4 (1.7) | 21.8 (3.0) | 19.0 (1.8) | 18.9 (2.3) | 23.4 (2.8) | 24.8 (5.7) |
| With a vegetarian / vegan* | 29.8 (1.2) | 39.2 (2.4) | 37.9 (3.5) | 28.3 (2.1) | 23.4 (2.8) | 17.0 (2.4) | 21.5 (6.1) |
| *Food attribute importance* | |  |  |  |  |  |  |
| Taste | 92.4 (0.4) | 91.5 (0.9) | 90.6 (1.8) | 92.6 (0.6) | 93.1 (0.9) | 94.4 (0.9) | 91.4 (2.4) |
| Health* | 58.9 (1.0) | 54.2 (1.9) | 66.9 (2.9) | 55.7 (1.7) | 66.1 (2.7) | 56.3 (2.4) | 71.6 (4.6) |
| Environment* | 49.3 (1.0) | 53.0 (2.0) | 60.0 (2.5) | 49.7 (1.8) | 47.4 (3.0) | 35.6 (2.6) | 42.7 (5.9) |
| Value | 78.4 (0.7) | 77.0 (1.3) | 77.6 (1.9) | 78.3 (1.2) | 80.0 (1.9) | 80.2 (1.8) | 79.1 (4.9) |
| Habit* | 47.9 (1.1) | 39.6 (1.9) | 42.1 (2.9) | 44.3 (1.9) | 57.7 (2.7) | 59.7 (2.7) | 74.4 (3.3) |
| *Voting behaviour in Australian Federal election 2022** | | | |  |  |  |  |
| Greens | 30.6% (0.8%) | 47.9% (1.9%) | 30.4% (2.4%) | 34.8% (1.6%) | 10.5% (1.0%) | 16.1% (1.4%) | 8.7% (1.7%) |
| Independent and others | 5.6% (0.2%) | 5.5% (0.4%) | 6.3% (0.7%) | 5.1% (0.3%) | 4.2% (0.4%) | 5.7% (0.6%) | 13.0% (2.4%) |
| Labor | 34.2% (0.9%) | 30.9% (1.7%) | 29.1% (2.3%) | 36.9% (1.7%) | 41.1% (2.5%) | 35.6% (2.5%) | 17.4% (3.0%) |
| Liberal-National Coalition | 8.2% (0.3%) | 1.2% (0.1%) | 6.3% (0.7%) | 5.6% (0.4%) | 14.7% (1.3%) | 17.2% (1.5%) | 26.1% (4.0%) |
| One Nation | 1.1% (0.0%) | 0.0% (0.0%) | 1.3% (0.1%) | 0.5% (0.0%) | 1.1% (0.1%) | 1.1% (0.1%) | 13.0% (2.4%) |
| I did not vote | 11.7% (0.4%) | 9.7% (0.7%) | 12.7% (1.2%) | 9.6% (0.6%) | 16.8% (1.4%) | 12.6% (1.2%) | 17.4% (3.0%) |
| Prefer not to say | 8.7% (0.3%) | 4.8% (0.4%) | 13.9% (1.3%) | 7.6% (0.5%) | 11.6% (1.1%) | 11.5% (1.1%) | 4.3% (0.9%) |
| *Personality* |  |  |  |  |  |  |  |
| Cares about environment* | 73.5 (0.8) | 78.9 (1.4) | 77.8 (2.3) | 73.5 (1.4) | 70.0 (2.3) | 65.2 (2.5) | 66.0 (5.2) |
| Cares about fitness* | 65.3 (1.0) | 62.5 (1.9) | 74.2 (2.3) | 61.5 (1.9) | 69.1 (2.8) | 66.0 (2.6) | 69.8 (4.5) |
| Cares about others’ opinion* | 53.8 (1.0) | 52.7 (2.0) | 45.2 (2.9) | 57.6 (1.8) | 52.6 (3.0) | 57.1 (2.9) | 49.6 (5.5) |
| Cares about health* | 74.0 (0.8) | 72.5 (1.6) | 81.2 (1.6) | 71.6 (1.6) | 74.2 (2.4) | 75.4 (2.2) | 74.8 (4.3) |
| Cares about animals | 78.0 (0.9) | 79.6 (1.9) | 82.6 (2.4) | 77.8 (1.6) | 78.8 (2.2) | 73.0 (2.7) | 67.8 (4.9) |
| Knows about plant-based meats* | 36.8 (1.0) | 47.9 (2.1) | 43.4 (3.0) | 33.3 (1.7) | 29.7 (2.6) | 26.4 (2.3) | 32.4 (5.1) |
| Knows about nutrition* | 55.6 (1.0) | 55.8 (1.9) | 64.9 (2.7) | 52.9 (1.8) | 55.8 (2.8) | 53.1 (2.5) | 53.0 (4.8) |
| Knows about climate change* | 60.6 (1.0) | 66.5 (1.8) | 63.6 (2.8) | 59.4 (1.6) | 55.8 (2.6) | 53.6 (2.8) | 65.3 (5.5) |
| Is open to new experiences* | 74.2 (0.8) | 75.4 (1.5) | 77.9 (2.0) | 73.9 (1.3) | 74.2 (2.1) | 70.5 (2.3) | 69.4 (4.4) |
| I*mportance of reducing meat consumption** | 60.3 (1.1) | 73.1 (1.5) | 69.3 (2.8) | 62.9 (1.6) | 47.3 (2.9) | 42.0 (2.8) | 38.7 (6.2) |

Note: * indicates significant difference at the .05 level.
